# Multi‐Modal Locomotion of *Caenorhabditis elegans* by Magnetic Reconfiguration of 3D Microtopography

**DOI:** 10.1002/advs.202203396

**Published:** 2022-10-31

**Authors:** Jeong Eun Park, Sunhee Yoon, Jisoo Jeon, Chae Ryean Kim, Saebohm Jhang, Tae‐Joon Jeon, Seung Goo Lee, Sun Min Kim, Jeong Jae Wie

**Affiliations:** ^1^ The Research Institute of Industrial Science Hanyang University Seoul 04763 Republic of Korea; ^2^ Program in Environmental and Polymer Engineering Inha University Incheon 22212 Republic of Korea; ^3^ Department of Biological Sciences and Bioengineering Inha University Incheon 22212 Republic of Korea; ^4^ Department of Chemistry University of Ulsan Ulsan 44610 Republic of Korea; ^5^ Department of Mechanical Engineering Inha University Incheon 22212 Republic of Korea; ^6^ Department of Organic and Nano Engineering Hanyang University Seoul 04763 Republic of Korea; ^7^ Human‐Tech Convergence Program Hanyang University Seoul 04763 Republic of Korea

**Keywords:** 3D microtopography, *Caenorhabditis elegans*, magnetic shape‐reconfiguration, microbarrier, multi‐modal locomotion

## Abstract

Miniaturized untethered soft robots are recently exploited to imitate multi‐modal curvilinear locomotion of living creatures that perceive change of surrounding environments. Herein, the use of *Caenorhabditis elegans* (*C. elegans*) is proposed as a microscale model capable of curvilinear locomotion with mechanosensing, controlled by magnetically reconfigured 3D microtopography. Static entropic microbarriers prevent *C. elegans* from randomly swimming with the omega turns and provide linear translational locomotion with velocity of ≈0.14 BL s^−1^. This velocity varies from ≈0.09 (for circumventing movement) to ≈0.46 (for climbing) BL s^−1^, depending on magnetic bending and twisting actuation coupled with assembly of microbarriers. Furthermore, different types of neuronal mutants prevent *C. elegans* from implementing certain locomotion modes, indicating the potential for investigating the correlation between neurons and mechanosensing functions. This strategy promotes a platform for the contactless manipulation of miniaturized biobots and initiates interdisciplinary research for investigating sensory neurons and human diseases.

## Graphical abstract

Locomotion of *Caenorhabditis elegans* is selectively guided via magnetically reconfigurable 3D microtopography. Upon magneto‐mechanical actuation including twisting actuation, bending actuation, and assembly of the microbarriers, locomotion of *C. elegans* converts from navigating to circumventing movement and climbing with minimum ≈0.09 BL s^−1^ and maximum ≈0.46 BL s^−1^ locomotion velocity, respectively.

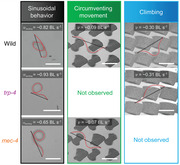

## 1 Introduction

Untethered miniaturized soft robots have recently been devised to perform difficult or delicate tasks for human beings, particularly in limited spaces influenced by external stimuli.^[^ ^1^ ^]^ To mimic the various locomotive behaviors of living organisms, the alignment of polymeric molecules or magnetic particles is programmed to achieve crawling,^[^ ^2^ ^]^ rolling,^[^ ^3^, ^4^ ^]^ swimming,^[^ ^5^, ^6^ ^]^ or jumping.^[^ ^7^ ^]^ Despite the progress in artificial soft robots, the performance of miniaturized soft robots is incomparable to that of living creatures capable of mechanosensing their surroundings and responding with complex curvilinear motility. As an alternative, living animals and insects have in recent times been adopted as biobots whose behavior is regulated by stimulating their nerve cells. Some examples include Sprague Dawley rat,^[^ ^8^, ^9^ ^]^ locust,^[^ ^10^ ^]^ beetle,^[^ ^11^ ^]^ and dragonfly^[^ ^12^ ^]^ under various stimuli, such as optical light,^[^ ^8^, ^10^, ^12^ ^]^ chemical fluids,^[^ ^9^ ^]^ and direct electric signals.^[^ ^11^ ^]^ However, these systems require the insertion of circuit chips and batteries, consequently limiting further miniaturization of biobots. Inserted batteries should be replaced regularly owing to their finite lifespans and temporary capacities. Furthermore, mistreatment issues have been raised with respect to ethical animal welfare,^[^ ^13^, ^14^ ^]^ immune rejection, and pain resulting from the insertion of foreign substances.

In this study, we propose contactless magnetic manipulation of 3D microtopographies to physically guide the complex locomotions of living animals. Instead of harmful surgeries or stimulation, this approach utilizes mechanosensing found in animals, which is an essential ability among living creatures to protect themselves from danger by sensing the applied stress, strain, substrate rigidity, and adhesiveness of their surrounding environments.^[^ ^15^ ^]^ We propose *Caenorhabditis elegans* as a microscale model mechanosensing system. The well‐differentiated nervous system of *C. elegans* renders it suitable for elucidating the mechanisms of mechanosensing and its relationship with underlying neuronal mutations.^[^ ^16^ ^]^ Since the discovery of connectome (a network of connections among neurons) in 2019,^[^ ^17^ ^]^ *C. elegans* has been recognized as the most reliable model of sensory neurons. It has been used in experiments to treat encephalopathy^[^ ^18^ ^]^ and aging diseases in humans^[^ ^19^, ^20^ ^]^ because its genetic similarity with humans reaches ∼ 60%.^[^ ^21^ ^]^ The reproducibility of the experiment is also confirmed in hermaphrodite *C. elegans* ^[^ ^22^, ^23^, ^24^ ^]^ using the organism's own sperm and eggs to reproduce generations with identical chromosomes.

The most notable locomotive characteristic of *C. elegans* is its sinusoidal behavior on an agar plate. It moves in random courses by changing its translational directionality through an omega‐shaped turn (called omega turn^[^ ^25^ ^]^) by rolling its body. Recently, modification of the natural behavior of *C. elegans* by paralyzing its nerve cells with ivermectin treatment has been reported. By locally irradiating the muscle cells of *C. elegans*, the crawling motion is guided in a specific direction.^[^ ^26^ ^]^ To achieve this guidance, the paralyzing process must be implemented, and complex software is necessary to guide motions of the worm. In lieu of the foregoing, with the introduction of a microfluidic channel into the agar plate environment, neurons in the head, body, and tail of *C. elegans* are stimulated.^[^ ^16^, ^27^, ^28^ ^]^ Although behavioral traits (i.e., preference for narrow spaces) have been identified, locomotion cannot be controlled dynamically using static microfluidic channels. For multi‐modal locomotion, a shape‐reconfigurable microsystem is necessary to introduce 3D dynamic microbarriers in *C. elegans*.

In this paper, we report multi‐modal locomotion of *C. elegans* physically guided by the magnetic shape reconfiguration of 3D microbarriers causing spatial confinement. The programmed locomotion of *C. elegans* includes linear translation, navigating movement, circumventing movement, and climbing, depending on the magnetic actuation modes of micropillars. Herein, the experimental environment was devised as a polydimethylsiloxane (PDMS) chamber filled with a buffer solution into which programmable microbarriers composed of PDMS composites were introduced. The periodically arranged micropillars were designed with a narrow *y*‐axial spacing of ≈50 µm to spatially confine *C. elegans* with a body width (BW) ≈50 µm. Conversely, the *x*‐axial spacing is wide at ≈ 210 µm, which is sufficient for sinusoidal behavior. Thus, *C. elegans* can perceive two different orthogonal linear pathways. For shape‐morphing of 3D microbarriers, magnetic micropillars are prepared by the inclusion of ferromagnetic iron particles in the PDMS matrix that are orthogonally or diagonally aligned with respect to the thickness of the micropillars. Under a linear external magnetic field, the micropillars undergo twisting actuation, bending actuation, or magnetic assembly to minimize magnetic interaction energy^[^ ^29^ ^]^ between the magnetic field and aligned particles. Before applying the magnetic field, the upright micropillars act as static barriers with simple microtopography. However, the magneto–mechanical actuations of the micropillars narrow the spacing between microbarriers. *C. elegans* perceives changes in the surrounding barriers and modifies locomotion and pathways to adapt to intricate 3D microtopographies via mechanosensing. Whether a neuronal mutant can perform this modified locomotion is employed as grounds to identify the type of neuronal mutant that cannot accommodate normal mechanosensing. We establish a novel platform for microscale biobots whose locomotion is controlled through a magnetic field system with the involvement of human engineering; however, their biological nature remains intact. This strategy is anticipated to aid in thoroughly exploring the mechanosensing locomotion of biobots^[^ ^30^, ^31^ ^]^ in an arbitrary environment for biomedical platforms and drug screening.^[^ ^32^, ^33^ ^]^

## 2 Results and Discussion

### 2.1 *C. elegans* Worm

**Figure 1:**
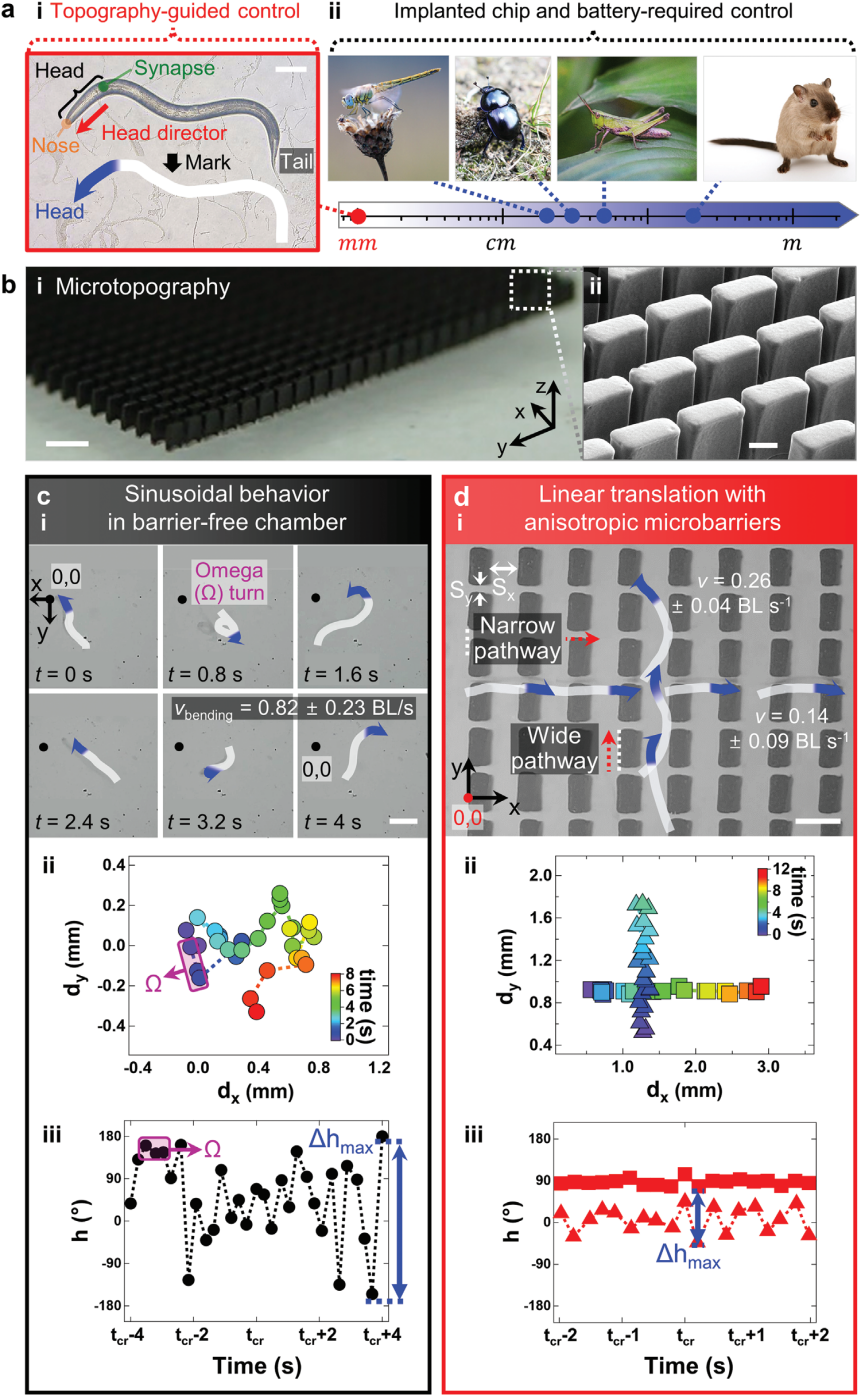
Biaxially controlled linear translation of *C. elegans* on environments of periodically arranged 3D microbarriers. a) Living creatures with artificially manipulated locomotion (from milimeter to meter). i) *C. elegans* (scale bar: 100 µm), ii) dragonfly, beetle, locust, and Sparague Dawley rat. b) Tilt‐view optical (scale bar: 500 µm) and scanning electron microscopy (scale bar: 200 µm) image of periodically arranged rectangular microbarriers. c) Random heading of *C. elegans* in bath without barriers. c‐i) Snapshot images, c‐ii) *x*‐displacement (*d_x_*), *y*‐displacement (*d*_y_), and c‐iii) head director (*h*) indicate sinusoidal behavior (scale bar: 200 µm). d) Biaxial linear translation of *C. elegans* at paths among microbarriers. d‐i) Optical image, graphs of d‐ii) *xy*‐coordinates and d‐iii) *h* oscillation denote linear translation in narrow (■) and wide (▲) pathways (scale bar: 300 µm).

To stimulate living worms while they remain intact, we propose a mechanism by which magnetically shape‐reconfigurable microtopographies trigger the multi‐modal motility of microscale *C. elegans*. The adult stage of wild type *C. elegans* is adopted as the target model in which sensory neurons sufficiently grow and normally function. In the adult stage, wild type *C. elegans* has mechanoreceptor neurons located in the body (e.g., ALM and AVM) and head (e.g., ASH and FLP).^[^ ^16^, ^34^ ^]^ Its body length (BL) is ≈ 1 mm, and BW ranges from 40 to 60 µm. We support the development of sub‐millimeter‐scale biobots as necessary advancement over previous biobots with scales ranging from meters to centimeters (**Figure** **1**a). We expect that the mechanosensing capability and adaptable nature of living *C. elegans* will be suitable for manipulation through physical guidance. In this study, the physical guidance was designed using microtopographies that magnetically shape‐reconfigure by a magnetic field harmless to living animals. However, typically, arguments for and against magnetotaxis have been discussed by many researchers. Although a study showed that *C. elegans* senses earth's magnetic field using their AFD neurons in the head,^[^ ^35^ ^]^ rebuttal processes consecutively followed.^[^ ^36^, ^37^ ^]^ In addition, a recent study reported that exposure to a magnetic field of 1 T for 48 h cannot change the behavior of *C. elegans*.^[^ ^38^ ^]^ As shown in Figure S1a–d, Supporting Information, as part of our experimental investigation, at barrier‐free chamber under linear magnetic field, *C. elegans* did not exhibit preferred directionality in the progress of sinusoidal behavior. After this confirmation, we observed and analyzed locomotion of *C. elegans* as it distinctively emerged from our magnetically shape‐reconfigurable 3D microtopographies. For visualization of transparent *C. elegans* on a transparent PDMS‐based chamber, their body shape was visually marked by a curvilinear arrow with a white body and blue head (Figure 1a‐i).

### 2.2 Static Microtopography With Upright Microbarriers

We placed PDMS/iron micropillar arrays on a PDMS‐based chamber to confine the pathway of *C. elegans* while preserving the nature of sinusoidal behavior (Figure 1b). Typical laboratory experiments are performed in agar plates or microchannels without specially designed obstacles. However, in this study, the environments were prepared with microbarriers to provide spatially limited stimulation to *C. elegans*. Rectangular micropillars were arranged in a rectangular lattice with a narrow *y*‐axial spacing of *S_y_* ≈ 50 µm and wide *x*‐axial spacing of *S_x_* ≈ 210 µm. Their thickness, *t*, width, *w*, and length, *l*, of the rectangular cross‐sections were ≈110, 250, and 350 µm, respectively. These dimensions were designed to induce high deformability of the micropillars utilizing shape anisotropy with aspect ratio of *w*/*t* ≈ 2.3 and *l*/*t* ≈ 3.2.

**Figure 2:**
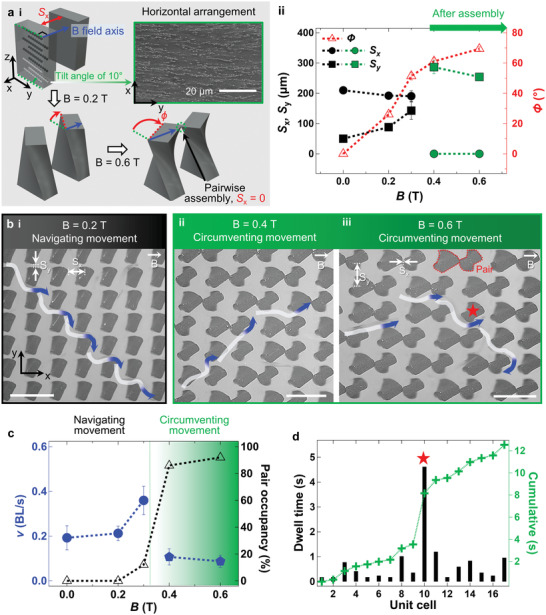
Circumventing movement on twisted and pairwise‐assembled micropillar arrays. a‐i) Mechanism of twisting/pairwise assembly caused by horizontal particle arrangements. a‐ii) Spacings *S_x_* and *S_y_* before (●,■) and after (●,■) assembly; twisting angle (*ϕ*) depending on *B*. b) Multi‐modal locomotion. b‐i) Navigating movement at *B* = 0.2 T, b‐ii) circumventing movement at *B* = 0.4 T, and b‐iii) 0.6 T (scale bars: 500 µm). c) Variation of *v* according to locomotion and pair occupancy at increased *B*. d) Dwell time in unit cell defined by Voronoi regimes. Longest dwell time point is indicated by ★.

**Figure 3:**
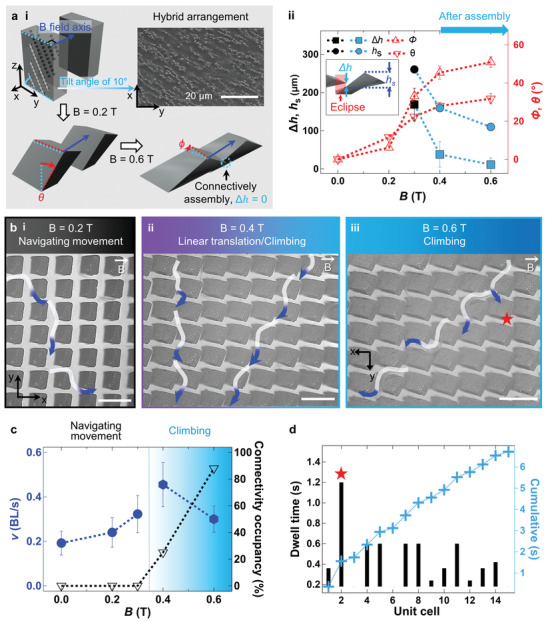
Climbing behaviors on twisted and bent micropillar arrays. a‐i) Mechanism of twisting/bending actuation/connective assembly caused by hybrid particle arrangements in horizontal and vertical axes. a‐ii) Height of step *h*_s_ and height difference, Δ*h*, before (●,■) and after (●,■) assembly, twisting angle (*ϕ*), and bending angle (*θ*) depending on *B*. b) Multi‐modal locomotion. b‐i) Navigating movement at *B* = 0.2 T, b‐ii) hybrid of linear translation and climbing at *B* = 0.4 T, and b‐iii) climbing at *B* = 0.6 T (scale bars: 500 µm). c) Variation of *v* according to locomotion and connectivity occupancy at increased *B*. d) Dwell time in unit cell defined by Voronoi regimes. Longest dwell time point is indicated by ★.

A design consisting of hundreds of micrometers was prepared through light‐assisted printing using a digital micromirror device (DMD)^[^ ^39^ ^]^ (Figure S2, Supporting Information). A positive master mold was fabricated using photomasking polyurethane acrylate (PUA) resin and then replicated into a PDMS negative mold. The negative mold was filled with PDMS/iron mixture through an evacuation process. Subsequently, a neat PDMS layer was prepared as a magnetically inert substrate and flexible hinge for the reversible magnetic actuation of pillars. To dynamically vary interpillar spacings, we employed the magnetically shape‐reconfigurable actuations of PDMS/iron micropillar arrays.^[^ ^40^, ^41^, ^42^ ^]^ Here, ferromagnetic iron particles (15 vol%) were arranged in chains within the PDMS matrix by placing the mixture‐filled mold between two permanent magnets. This particle arrangement generated directional magnetic actuations (twisting and bending) with assemblies (pairwise and connective) of the micropillars. The detailed mechanisms for the magnetic systems are described in **Figures** **2** and **3**. Through these magnetic systems non‐destructive to a living worm, shape‐reconfigured 3D microtopography provides physical guidance for the pathway of the worm without suppressing their natural sinusoidal behavior.

### 2.3 Linear Translation With Sinusoidal Behavior

Different from sinusoidal behavior in a barrier‐free chamber (Figure 1c), *C. elegans* exhibits two linear translations in an environment with periodically arranged upright microbarriers (Figure 1d). The static microbarriers have the same cross‐sectional areas along the *z*‐axis, providing uniform spatial confinement regardless of their position along the z‐axis. To compare and analyze the locomotion of *C. elegans*, barrier‐free sinusoidal behavior is described by the fact that head director (*h*) is not oriented toward a particular direction (i.e., random pathway) (Figure 1c‐i). Here, *h* is defined as the direction from the pharyngeal grinder toward the nose (Figure S3a, Supporting Information). Zero *h* represents the upward direction (positive in *y*‐axis, i.e., +y direction); and +*h* and −*h* correspond to the clockwise and counterclockwise directions, respectively (Figure S2b, Supporting Information). Furthermore, the omega turn (Ω) for switching directionality in a random pathway is depicted in overlapped *xy*‐coordinates (Figure 1c‐ii). The sinusoidal characteristic is denoted by the oscillation of *h* at a critical time (*t*_cr_) required to observe arbitrary movement (Figure 1c‐iii). The relatively large oscillation of *h* is quantified by the maximum difference between two head directors (Δ*h*_max_), that is, ≈350°. Conversely, with rectangular microbarriers, highly reduced Δ*h*_max_ appeared in the linear translational locomotion of *C. elegans* (Figure 1d‐i; Figure S4 and Video S1, Supporting Information) that predominated the diagonally headed locomotion. The Δ*h*_max_ values were ≈36° and ≈88° as neurons in the head and body of *C. elegans* sensed narrow and wide interbarrier spacings, respectively (Figure 1d‐iii). The two orthogonal linear pathways were observed in the *xy*‐coordinates (denoted by square [*x*‐axis] and triangular [*y*‐axis] symbols (Figure 1d‐ii). For these two cases, the Δ*h*_max_ value of linear translation in wide interbarrier spacings is 2.4 times larger than that in narrow interbarrier spacings (Table S1, Supporting Information); the locomotion velocity (*v*) is comparable. This *v* was an average measured by examining five *C. elegans* worms that locomote for distances longer than at least two body lengths. A lower standard deviation (stdev) of the average *v* implies that the locomotion is more reproducible with physical guidance. For the two different linear translations, the wide pathway showed a faster *v* of 0.26 BL s^−1^ and a lower stdev of 0.04 BL s^−1^, when compared to the narrow one with *v* of 0.14 BL s^−1^ and stdev of 0.09 BL s^−1^. As a result, perodically arranged anisotropic microbarriers provide two different linear pathways for *C. elegans* that perceive 3D environments via mechanosensing.

### 2.4 3D Microtopography by Magnetically Twisted and Pairwise‐Assembled Micropillar Arrays

Under a linear external magnetic field (Figure S5a,b, Supporting Information), complex 3D microtopography was programmed by twisting actuation and pairwise assembly (Video S2, Supporting Information), which modified interbarrier spacings (Figure 2a). For unidirectional twisting actuation, the arrangement of iron particles in the PDMS matrix was designed to have a pre‐tilt of 10° with respect to the width of the pillar, as indicated with the green dotted line (**…**) (Figure 2a‐i; Figure S2, Supporting Information). When a magnetic field (**→**) orthogonal to the horizontal particle arrangement is applied, the micropillars undergo twisting actuation with a controllable twisting angle (*ϕ*) according to magnetic flux density (*B*) (Figure 2a‐ii; Figure S6a–d and Table S2, Supporting Information). At *B* = 0.3 T, the micropillars twist with *ϕ* ≈ 51.4°; *S_x_* narrows from 210 to 190.7 µm whereas *S_y_* widens from ≈50 to ≈142.6 µm, as shown in Figure 2a‐ii and Table S3 in the Supporting Information, implying an increase in magnetic attraction among adjacent lateral micropillars. The magnetic polarities of iron particles are arranged along the external magnetic field, and the magnetization value of micropillars increases with *B*. As a result, the opposite edges of twisted adjacent pillar tops are self‐assembled in pairs via quadrupolar interactions. Here, design of *S_x_* smaller than the *w* of micropillar enables this pairwise assembly even with *ϕ* of ≈51.4° lower than 90°. The number of pairwise‐assembled micropillars is quantified as pair occupancy, and this pairwise assembly becomes dominant when *B* exceeds 0.4 T (Figure 2c). Twisting actuation in conjunction with the pairwise assembly of the microbarriers is utilized to guide the multi‐modal locomotion of *C. elegans* using shape‐reconfigurable microtopography depending on *B*.

### 2.5 Navigating and Circumventing Movements on Maze‐Like Microtopography

*C. elegans* demonstrated navigating and circumventing locomotion on magnetically reconfigured maze‐like 3D microtopography (Figure 2b–d; Video S3, Supporting Information). As mentioned, the microtopography can be tuned on demand according to *B*, which modifies the interbarrier spacing through twisting actuation as well as pairwise assembly. *C. elegans* moves forward by sensing wide *S_y_* spacings among twisted microbarriers using mechanosensory neurons located in its head up to *B* = 0.3 T. This type of locomotion that searches for pathways is called navigating movement (Figure 2b; Figure S7a–c, Supporting Information). *C. elegans* passes through spacings among twisted microbarriers in a streamlined manner with a trajectory (Figure 2b‐i; Figures S8a,b and S9a,b, Supporting Information). The living *C. elegans* adapts to the changed microtopography where *S_y_* widens from ∼ 50 to ∼ 142.6 µm, and *v* increases accordingly from ∼ 0.19 to ∼ 0.36 BL s^−1^ (Figure 2c; Figure S7a, Supporting Information).

However, beyond *B* = 0.4 T, the circumventing movement appears to deviate from the maze‐like 3D microtopography in which *S_x_* becomes zero through pairwise assembly (Figure 2b‐ii,iii; Figures S9c,d and S10a–d, Supporting Information). For the zero *S_x_*, the gap between assembled microbarriers is blocked at the top and narrowed at the bottom, as observed by side view images in Figure S6c‐ii,d‐ii, Supporting Information. Half of this gap has ∼ 142.1 µm height and ∼ 40.4 µm width, smaller than ∼ 155.6 µm head length and 40–60 µm BW of *C. elegans*, respectively. Accordingly, *C. elegans* considers this half of gap generated by the pairwise‐assembled microbarriers as an impassable space and circumvents the microbarriers. This locomotion type is called circumventing movement, and its *v* was significantly lower than that of the navigating movement (Figure 2c; Table S5, Supporting Information). With the circumventing movement, the value of *v* decreases from ∼ 0.36 to ∼ 0.11 and ∼ 0.09 BL s^−1^ as *B* increases from 0.3 to 0.4 and 0.6 T, respectively; the occupancy of pairwise‐assembled microbarriers increases from 12% to 86% and 96%, respectively. Here, stdev of *v* also decreases from 0.06 to 0.04 and 0.03 BL s^−1^, respectively, implying that circumventing movement is the most reproducible at *B* = 0.6 T. Note that the dwell time for *C. elegans* at each microbarrier was delayed in the intricate pathways at *B* = 0.6 T.

The dwell time in a single unit cell was also analyzed to determine differences in spatial confinements imposed on *C. elegans* by pairwise‐assembled and single microbarriers (Figure 2d; Figures S8c,d and S10b, Supporting Information). Here, the unit cell is defined by Voronoi analysis and indicates a region separated by perpendicular bisectors^[^ ^43^, ^44^ ^]^ through which *C. elegans* passes. In the case of the single microbarrier surrounded by pairwise‐assembled microbarriers, the unit cell has the narrowest *S_x_* value of ∼ 82 µm, resulting in the longest dwell time (★) for *C. elegans* (Figure 2b‐iii). The measured average dwell time for circumventing movement is 0.7 s, increasing from 0.5 s of the navigating movement (at *B* = 0.2 T) (Figure S8c,d, Supporting Information). Furthermore, note that statistical data examining the number of locomotion modes performed by *C. elegans* is included in Figure S11, Supporting Information. A probability (*p*) provides which locomotion mode is best physically guided at specific magnetic field conditions. For example, as shown in Figure S11b, Supporting Information, navigating and circumventing movements occur the most at *B* = 0.3 and 0.6 T, respectively. For navigating movement, *p* increases from 65.1% to 88.8% with twisting angle increasing from 0° to ∼ 51.4° at *B* below 0.3 T. In addition, *p* of the circumventing movement increases from 16.0% to 26.7% owing to the pair occupancy increase from 86% to 96% at *B* below 0.6 T. These analyses of *v*, dwell time, and *p* demonstrate that navigating and circumventing movements of *C. elegans* can be controlled as necessary by the reconfigurable spacings among the microbarriers via magnetic twisting/pairwise assembly mode.

### 2.6 3D Microtopography by Magnetically Twisted, Bent, and Connectively Assembled Micropillar Arrays

The microtopography was designed to have magnetic actuations of twisting, bending, and connective assembly under a linear external magnetic field (Figure 3a‐i; Video S2, Supporting Information). The 3D magneto–mechanical actuation modifies not only *S_x_* and *S_y_* but also the height (*h*) of the twisted and bent micropillars (Figure 3a‐ii; Figure S12a–h, Supporting Information). The iron particles were arranged diagonally (i.e., 45° or [211] plane) in the micropillar with a 10° pre‐tilt with respect to the width of the pillar as indicated with the sky blue dotted line (**…**) (Figure 3a‐i; Figure S2, Supporting Information). Thus, micropillars demonstrate complex actuations with both twisting and bending when a linear magnetic field (**→**) up to 0.6 T is applied. As *B* increases, *h* decreases because of the large bending actuation coupled with twisting (Figure S12h, Supporting Information). To quantify these morphological changes of micropillars, we also measured *S_x_*, *S_y_*, *h*, *ϕ*, and *θ* (bending angle) (Figure S12e–h and Tables S2–S4, Supporting Information).

Nearby micropillars are eclipsed owing to large bending actuations with *θ* ≈ 33.2° at *B* = 0.3 T, as shown in the top–down image view in Figure S12c‐i, Supporting Information. The *z*‐axial interval among the eclipsed micropillars is defined by the height difference (Δ*h*) (determined by vertically joining the edges of lower pillar top and adjacent pillar side) (Figure 3a‐ii; Figure S12c,d, Supporting Information). As *B* increases from 0.3 to 0.4 T, further twisting and bending actuations narrow *S_x_* from ∼ 94.6 to ∼ 26.8 µm and decrease Δ*h* from ∼ 168.7 to ∼ 37.8 µm (Figure S12g–h, Supporting Information). With increased magnetization values under a magnetic field, the neighboring dipolar micropillars self‐assemble by quadrupolar attractions (Figure 3a‐i; Figure S12d, Supporting Information). The edges of the lower pillar top and adjacent pillar side are in contact with a long‐range order; this is called connective assembly. The number of connectively assembled micropillars was quantified as connectivity occupancy (Figure 3c). As *B* increases from 0.4 to 0.6 T, connectivity occupancy increases from 25% to 88%, and Δ*h* and *θ* reach ∼ 11.4 µm and ∼ 51.0°, respectively. This magnetically reconfigured 3D microtopography selectively triggers the multi‐modal locomotion of *C. elegans* depending on changes in height, twisting/bending angle, spacing, and connectivity of actuated microbarriers under the magnetic field.

### 2.7 Navigating Movement and Climbing in Stair‐Like Microtopography

Climbing is distinctively demonstrated in 3D microtopography where twisted and bent microbarriers act as microsteps (Figure 3b–d; Video S4, Supporting Information). At *B* = 0.2 T, navigating movement emerges on the microbarriers with a relatively low *ϕ* value of ∼ 11.6° and *θ* of ∼ 6.2° (Figure 3b‐i; Figure S13a,b, Supporting Information). For the twisting actuation with increased *ϕ* value of ∼ 22.4° at *B* = 0.3 T, *S_x_* narrows from ∼ 210 to ∼ 94.6 µm, whereas *S_y_* widens from ∼ 50 to ∼ 116.3 µm. The magnitude of *v* in the navigating movement increases from ∼ 0.19 to ∼ 0.32 BL s^−1^ (Figure 3c); climbing also appears with navigating movement on the stair‐like 3D microtopography (Figure S13c,d, Supporting Information). *C. elegans* begins to step on the eclipsed bent microbarriers with increased *θ* value of ∼ 33.2°, and *z*‐axial intervals are defined as Δ*h* ≈ 168.7 µm. Here, the measured height of the step (*h*_s_) was ∼ 260.8 µm (obtained by vertically joining two edges of the upper pillar top and adjacent pillar side) (Figure S12c‐ii, Supporting Information). The quantified morphological indicators, Δ*h* and *h*_s_, are required for climbing locomotion. For example, as *B* increases from 0.3 to 0.4 T, climbing predominates navigating movement because the microsteps become smaller as *h*_s_ decreases from ∼ 260.8 to ∼ 159.6 µm. *C. elegans* considers twisted and bent microbarriers as microsteps that facilitate the climbing because its ∼ 155.6 µm head length approximates *h*_s_ ≈ 159.6 µm. Connective assembly also occurs at *B* exceeding 0.4 T. Here, the climbing speed, *v*, reaches 0.46 BL s^−1^ with stdev of 0.10 BL s^−1^ and then decreases to 0.30 BL s^−1^ with stdev of 0.13 BL s^−1^ as *B* increases to 0.6 T.This decrease in *v* is attributed to an *h*_s_ value of ∼ 101.0 µm, which is lower than the ∼ 155.6 µm head length of *C. elegans*. We also consider *v* as inversely related to connectivity occupancy, as shown in Figure 3c. For statistical analysis, measured *p* of climbing behavior also rather decreases from 39.6% to 30.5% as *B* increases from 0.4 to 0.6 T (Figure S11c, Supporting Information). This is because *h*_s_ at *B* = 0.4 T provides *C. elegans* with optimal microsteps to climb rather than *B* = 0.6 T, as mentioned earlier. In addition, *p* of sinusoidal behavior increases from 8.5% to 29.7% and 48.6% because *h* decreases from ∼ 329.4 to ∼ 305.3 and ∼ 259.7 µm as *B* increases from 0.3 to 0.4 and 0.6 T, respectively (Figure S12f,h, Supporting Information). Especially, at *B* = 0.6 T, the microbarriers with the lowest height *h* induce sinusoidal behavior with the highest *p* because of a swimmable spatial margin above the microtopography. Finally, the dwell time for climbing at *B* = 0.6 T is further analyzed by considering connectively assembled microbarriers in a row as a single unit cell (Figure S14b, Supporting Information). *C. elegans* has the longest dwell time (★) in the early stages of climbing before acclimating to the surrounding environment with a high connectivity occupancy of 88% (Figure 3b‐iii,c,d).

Furthermore, in addition to climbing, we also observed linear translation with the *xz*‐plane constricting the body of *C. elegans* among largely twisted and bent microbarriers (Figure 3b‐ii; Figure S15a–d, Supporting Information). The locomotion distinctively emerges in narrowed *y*‐axial pathways at *B* = 0.3 and 0.4 T, where twisted and bent microbarriers with small *S_x_* values (e.g., ∼ 26.8 µm at 0.4 T) act as trap‐like microtopography for *C. elegans*. Velocity *v* increases to ∼ 0.27 BL s^−1^ (with low sinusoidal oscillation depicted by the *xy*‐coordinates) at *B* = 0.3 T compared with *v* of ∼ 0.22 BL s^−1^ at *B* = 0.4 T. We confirmed that *C. elegans* exhibits unprecedented climbing on a stair‐like 3D microtopography, magnetically generated by twisting and bending actuation.

### 2.8 Behavioral Features of Mutants and Wild Type *C. elegans*

**Figure 4:**
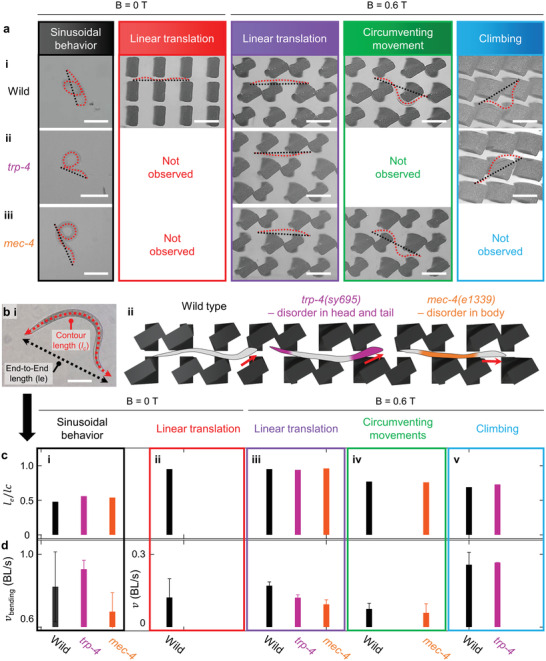
Comparision of length ratio, *l*_e_/*l*_c_, according to mechanosensing ability of wild type, head/tail mutant (*trp‐4*), and body mutant (*mec‐4*). a) Snapshot images of sinusoidal behavior, linear translation in narrow spacings, and circumventing movement and climbing behavior of a‐i) wild type, a‐ii) head/tail mutant (*trp‐4(sy695)*), and a‐iii) body mutant (*mec‐4(e1339)*) (scale bars: 300 µm). b‐i) Contour length, *l*_c_, and end‐to‐end length, *l*_e_, are indicated by red and black dot lines, respectively (scale bar: 200 µm). b‐ii) Schematic illustration of strain with disorder in head/tail (*trp‐4(sy695)*) (magenta) and body (*mec‐4(e1339)*) (orange). c) Length ratio, *l*_e_/*l*_c_, and d) bending velocity, *v*_bending_, in c‐i) sinusoidal behavior, locomotion velocity, *v*, in c‐ii,iii) linear translation, c‐iv) circumventing movement, and c‐v) climbing of wild type (black), head/tail mutant (*trp‐4(sy695)*) (magenta), and body mutant (*mec‐4(e1339)*) (orange).

*C. elegans* mutants, *trp‐4(sy695)* and *mec‐4(e1339)*, were employed to investigate behavioral features related to the mechanosensing ability that differs from that of the wild type (**Figure** **4**a). To develop treatment therapies for human genetic diseases,^[^ ^45^, ^46^ ^]^ mutants have been actively investigated with respect to mechanosensing systems that vary according to the individual gene disorder. In particular, two genotypes with disorders in the head/tail (*trp‐4(sy695*)) and body (*mec‐4(e1339)*) were adopted for comparison with the wild type (N2) (Figure 4b‐ii). The head/tail mutant *trp‐4(sy695)* on maze‐like microtopography is expected not to attain the circumventing movement that requires utilizing the head of *C. elegans* to find a path. Conversely, the body mutant *mec‐4(e1339)* in stair‐like microtopography is expected to have difficulty in climbing as this requires body neurons. The experiment verifies these assumptions. After describing the detailed features of each strain, explanations are presented in the next section.

Strain *trp‐4(sy695)* is a mutant in the *trp‐4* protein that is intensively expressed in the DVA, DVC, and dopaminergic neurons related to anterior and posterior touch, as opposed to the intact neuron in body. The representative features of *trp‐4(sy695)* include large oscillation amplitude with a relatively fat and long phenotype compared with the wild type at identical stages.^[^ ^47^ ^]^ For example, *trp‐4(sy695)* at the adult stage has a BL ≈ 1.2 mm and BW ≈ 77.4 µm (Figure S16a,c, Supporting Information), which are larger than those of the wild type at the same stage with BL ≈ 1.1 mm and BW ranging from 40 to 60 µm. Accordingly, in this study, we adopt *trp‐4(sy695)* at a young adult stage with BL ≈ 1.1 mm and BW in the range of 40 to 60 µm. This BW dimension, similar to the interbarrier spacing of ∼ 50 µm in our microtopography, allows *C. elegans* to avoid entrapment among the static microbarriers. Furthermore, the bending velocity (*v*_bending_) for sinusoidal behavior is compared in a barrier‐free chamber by observing the c‐shape bending transition of *C. elegans*, as shown in Figure S16b,d, Supporting Information. The bending velocity of the head/tail mutant *trp‐4(sy695)* is faster^[^ ^48^ ^]^ (*v*_bending_ ≈ 0.93 BL s^−1^) than that of the wild type (*v*_bending_ ≈ 0.82 BL s^−1^) (Table S6, Supporting Information).

Meanwhile, the body mutant *mec‐4(e1339)* has a genetic mutation in the *mec‐4* gene that encodes a membrane protein required to sense soft mechanical stimuli applied to the body wall, contrary to the intact neuron in head/tail. It has lower sensitivity to body contact, and its bending velocity is lower^[^ ^49^ ^]^ (*v*_bending_ ≈ 0.65 BL s^−1^) than that of the wild type (*v*_bending_ ≈ 0.82 BL s^−1^). The target stages for synchronization were chosen as *mec‐4(e1339)* at a young adult stage with BL ≈ 1.0 mm and BW ranging from 40 to 60 µm, similar to the case of young adult *trp‐4(sy695)*) and adult wild type. This BW dimension excludes undesired changes in locomotion resulting from difference in body dimensions among the three strains at the same stage. These strains are depicted in magenta and orange in Figure 4b‐ii to describe the gene disorder sites of the *trp‐4* and *mec‐4* mutants, respectively. The locomotion capacity of the wild type is compared with that of *trp‐4(sy695)* with a disorder in the head/tail and *mec‐4(e1339)* with a disorder in the body.

### 2.9 Selective Locomotion on 3D Microtopography Depending on Gene Disorder

To determine which neurons are necessary to accomplish distinct locomotions in magnetically shape‐reconfigurable microtopographies, a specific mechanism is demonstrated in this study (Figure 4a–c). In programmed environments, the three strains (wild [i], head/tail mutant *trp‐4* [ii], and body mutant *mec‐4* [iii]) selectively exhibited different locomotions due to their individual gene disorders. Notable differences between the head/tail mutant *trp‐4(sy695)* and body mutant *mec‐4(e1339)* were identified in circumventing movement and climbing, respectively (Figure 4a; Figures S17a,b and S18a,d, Supporting Information). Neurons in the body and head/tail are verified to be essential for circumventing movement and climbing, respectively. Furthermore, sinusoidal behavior^[^ ^45^, ^46^ ^]^ (enclosed in black) and linear translation on twisted and pairwise‐assembled microtopography (enclosed in purple) are possible in all the three strains despite abnormalities in the head/tail or body of *C. elegans* (Figure S19a,b and Video S1, Supporting Information). In particular, in the case of twisting/pairwise assembly mode, *C. elegans* moves forward linearly among actuated pillar tops where *S_y_* widens from ∼ 50 to ∼ 79.6 µm via mechanosensing using respective neurons of strains (Video S5, Supporting Information). However, the linear translation among upright barriers with narrow *S_y_* value of ∼ 50 µm (enclosed in red) requires intact neurons from the head to the body and tail. The feasible locomotion of the strains is summarized in a 2D map (Figure S20, Supporting Information) identifying neurons required for the specific locomotion.

### 2.10 BL in Multi‐Modal Locomotion

Quantitative indicators were explored to distinguish the specific locomotion and behavioral features caused by the gene disorders of head/tail mutant *trp‐4(sy695)* and body mutant *mec‐4(e1339)* and compare them with those of the wild type (Figure 4b‐d). Among the features, BL varies according to the genotype and locomotive behavior. The contour length (*l*_c_) signifies a physically possible extension of *C. elegans* body. At a given *l*_c_, the small end‐to‐end length (*l*_e_) implies sinusoidal and streamlined attributes in the locomotion of *C. elegans*. To normalize BL for classifying the three strains, the linearity of *C. elegans* is defined based on a length ratio of *l*_e_/*l*_c_ during locomotion. An *l*_e_/*l*_c_ approaching 1 (e.g., 0.94–0.96) represents linear translation because *C. elegans* cannot bend its body when spatially confined among the static as well as actuated microbarriers (Figure 4c‐ii,iii; Table S6, Supporting Information). The relationship *l*_e_ ≈ *l*_c_ is a result that has not been previously reported; the only related previous study is on rigor mortis in *C. elegans*. Straight body morphology resulting from the muscle contraction of the body wall with the release of Ca^2+^ ions and a drop of adenosine triphosphate has only been observed in dead *C. elegans*.^[^ ^50^ ^]^ In addition, for sinusoidal behavior, *C. elegans* folds its body in half with the omega turn to change the direction of progress. This yields *l*_e_/*l*_c_ in the range of 0.5–0.6, which does not considerably vary regardless of the genotype (Figure 4c‐i). Last, *C. elegans* moves with a streamlined body morphology for circumventing movement and climbing at *B* = 0.6 T, resulting in *l*_e_/*l*_c_ between 0.5 and 1, specifically 0.8 and 0.7, respectively (Figure 4c‐iv,v). These results indicate that the BL of *C. elegans* can be considered a useful indicator to identify locomotion features regardless of the genotype.

### 2.11 Locomotion Velocity of Three Different Strains

The locomotion velocity, *v*, was analyzed to explore the capacity of the three *C. elegans* strains for multi‐modal locomotion (Figure 4d). For sinusoidal behaviors, the head/tail mutant *trp‐4(sy695)* exhibits fast *v*_bending_ with a large swimming amplitude^[^ ^47^ ^]^ and random progress direction. As shown in Figure 4d‐i, the magnitude of bending velocity on flat substrates reveals that the head/tail mutant *trp‐4(sy695)* is the fastest (*v*_bending_ ≈ 0.93 BL s^−1^) compared with the wild type (*v*_bending_ ≈ 0.82 BL s^−1^) and body mutant *mec‐4(e1339)* (*v*_bending_ ≈ 0.65 BL s^−1^). However, for *v* to be high in microtopography‐induced locomotion, particularly at *B* = 0.6 T, the neurons in the head/tail and body should be intact. For linear translation (Figure 4d‐iii), the velocity of the wild type is ∼ 0.20 BL s^−1^; this is ∼ 1.41 times faster than that of head/tail mutant *trp‐4(sy695)* (*v* ≈ 0.14 BL s^−1^), despite comparable head oscillations of the two strains (Figure S19a‐i,ii,b‐i,ii, Supporting Information). This result implies that our magnetically shape‐reconfigured 3D microtopography can be applied as a platform for modifying behavioral features (e.g., body length morphology and velocity) of *C. elegans*. The examination of modified behavioral traits in *C. elegans* is also anticipated to aid in the development of a drug screening platform. After drug treatment, mutants will be tested for locomotions physically guided by our microtopography.

### 2.12 Present Limitation and Future Research

For exploitation of *C. elegans* as sub‐millimeter scale biobots or model of drug screening test, this study should be developed further. In the scope of this paper, *C. elegans* cannot solve difficult problems inside working spaces not yet accessible to humans. In addition, dimensional mismatch between body of *C. elegans* and microtopography can limit the physical guidance caused by a mechanosensing system. For example, the circumventing movement mode is achieved in the case that the gap between twisted and pairwise‐assembled microbarriers is smaller than BW and head length of *C. elegans*. For the climbing behavior mode, height of steps between twisted and bent microbarriers must be similar or smaller than the head length of *C. elegans*. Last, while performing multi‐modal locomotion, the worm cannot control directionality of progress, a requisite for fully functional biobots.

To overcome these unresolved limitations, a future study is expected to include simulations and deep learning to scrutinize complicated neuronal responses for the multi‐modal locomotion of *C. elegans*. Furthermore, a systematic design with repeated demonstrations may be introduced into the 3D microtopography to fulfill dimensions required for *C. elegans* with different sizes in populations. Last, additional stimuli can also be introduced to attract living worms and determine the directionality of progress. As an example, chemotaxis or electrotaxis can be further applied to the platform of magnetically shape‐reconfigurable 3D microtopographies demonstrated in this paper. The taxis behaviors indicate that *C. elegans* orients in a certain direction to chase prey while avoiding toxic substances or to attenuate undesired impulses of ASJ and ASH neurons.^[^ ^51^ ^]^ In exploring these areas further, we aim to improve the ability to control multi‐modal locomotion of *C. elegans* and develop applications such as drug screening in therapeutic technologies for the healthcare industry. In previous study regarding the drug screening, drug effect was investigated by treating a specific drug and examining recovered electrotaxis ability of *C. elegans* with a neuronal disorder. This strategy can be similarly applied to our study by comparing the physically guided locomotion abilities of wild and mutant strains after drug treatment. Systematic repetition of this process will lead to determining the most effective drug for treating neurological diseases. With the assistance of computational simulations, future studies would consequently result in the advancement of drug screening platforms.

## 3 Conclusion

In this study, the living microscale worm is investigated without destroying or oppressing their nature; the multi‐modal locomotion of *C. elegan*s by mechanosensing a magnetically shape‐reconfigured 3D microtopography is reported. Although *C. elegans* is widely known to have sinusoidal behavior with random directions, static micropillar arrays here act as microbarriers, guiding *C. elegans* to implement linear translation. Subsequently, we programmed 3D microtopography to achieve magneto–mechanical actuations including twisting, bending, and assembly. The modified 3D microtopography induces multi‐modal locomotion, including linear translation, navigating movement, circumventing movement, and climbing. Here, a key factor in selecting locomotion on demand is the regulation of interpillar spacings and pillar height according to magnetic flux density. Interpillar spacings are largely narrowed with twisting actuation and pairwise assembly, converting the locomotion mode from navigating to circumventing movement and decreasing locomotion velocity from ∼ 0.21 to ∼ 0.09 BL s^−1^. The lowest velocity, ∼ 0.09 BL s^−1^, was attributed to the intricate maze‐like pathways. In contrast, along with an increase in locomotion velocity (from ∼ 0.24 to ∼ 0.46 BL s^−1^), stair‐like topography prepared on twisted and bent microbarriers transforms the locomotion mode from navigating movement to climbing. For climbing with the fastest velocity of ∼ 0.46 BL s^−1^, we need to avoid connective assembly in microbarriers causing height of step lower than the head length of *C. elegans*. Control of twisting and bending actuations allows *C. elegans* to mechanosense the stair and facilitate moving forward through the climbing. In addition, we examined which locomotion was possible despite gene disorders in specific neurons by comparing the head/tail mutant *trp‐4(sy695)* and body mutant *mec‐4(e1339)* with the wild type. By determining locomotion velocity, we identify the effects of neuron disorders on the capacity for locomotion. As a result, the neurons in the head/tail and body are required to be intact for fast linear translation, circumventing movement, and climbing locomotion induced by 3D microtopography. The foregoing is contrary to the typical fact that the head/tail mutant *trp‐4(sy695)* exhibits faster sinusoidal behavior with a large oscillation amplitude in a barrier‐free chamber, when compared with the wild type. In the field of biomedical technologies, a dynamically deformed 3D platform could be developed to explore living *C. elegans* applied for drug screening in the biomedical discipline.

## 4 Experimental Section

### Preparation of Positive PUA Mold

The process for fabricating positive PUA mold is shown in Figure S2, Supporting Information. The glass substrate was placed in 1‐m sodium hydroxide aqueous solution for 1 h, followed by rinsing with deionized (DI) water. The glass substrate was sequentially treated with plasma cleaner (Harrick Plasma PDC‐32G‐2) for 30 s and then coated with a solution of 5% (v/v) 3‐(trimethoxysilyl) propyl acrylate (Sigma–Aldrich) in ethanol for 1 h. After washing with ethanol, the acrylate‐coated substrates were annealed at 80 °C for 15 min. The other glass substrate was cleaned using plasma cleaner and placed in a vacuum desiccator with 30‐µL trichloro(1H,1H,2H,2H‐perfluorooctyl) silane (Sigma–Aldrich) for 2 h. After washing with ethanol, ultraviolet (UV)‐curable PUA (MINS 311 RM, Minuta Tech) resin was dispensed onto the acrylate‐coated glass substrate to act as a supporting backplane. Then, the PUA resin was covered with the hydrophobic‐coated glass substrate with a thickness regulated by two spacers and pressed with a relatively weak pressure of ∼ 10 Pa. The sample was exposed to UV light generated by a UV light‐emitting diode source (Lumen 200, Prior) and briefly reflected by DMDs (Andor, Mosaic3) for 7 s. After curing the PUA, the hydrophobic‐coated glass and spacers were removed, washed with DI water and ethanol, and then dried.

### Preparation of Negative PDMS Mold

A positive PUA master mold was treated by hydrophobic coating using 30 µL of vaporized trichloro (1H,1H,2H,2H‐perfluorooctyl) silane (Sigma–Aldrich) in a vacuum desiccator for 2 h, preceded by plasma treatment for 30 s. Sylgard 184 PDMS (Dow Corning) was utilized to replicate the positive PUA mold. A mixture of the PDMS prepolymer and curing agent (10:1 w/w) was poured onto the PUA mold and then cured at 80 °C for 3 h. Subsequently, the cured PDMS mold was peeled off from the PUA mold.

### Fabrication of Magnetically Reconfigurable Microarrays

The negative mold was covered with a polymeric composite mixture containing PDMS resin and iron particles (HQ grade, BASF). Iron particles were loaded into the polymeric composite at 15 vol%, and evacuation was performed in a vacuum chamber to completely fill the mold with composites. The iron particles were chain‐like arranged by a linear magnetic field generated by two permanent magnets (N35 grade, Kingkong magnets)^[^ ^40^, ^41^ ^]^ with a customized stage. Particle arrangements were delicately manipulated in the horizontal axis (for the twisting/pairwise assembly mode) and in the hybrid axis of the horizontal and vertical axes (for the twisting/bending actuation mode).^[^ ^40^ ^]^ Subsequently, pre‐curing was performed at 90 °C for 5 min to immobilize the iron particles within the PDMS matrix. The PDMS resin mixture was covered again on the prepared mold to produce a magnetically inert substrate. Full curing was performed at 90 °C for 1 h. Then, microarrays were harvested from the mold.

### *C. elegans* Strains

All strains of *C. elegans* were obtained from the *Caenorhabditis* Genetic Center and cultured at 20 °C using nematode growth medium (NGM) plates with *Escherichia coli* OP50 as food. In all experiments, M9 buffer (KH_2_PO_4_, Na_2_HPO_4_, NaCl, and 1 m MgSO_4_) was used to collect and load the worms. Worm synchronization was performed according to a common protocol using a hypochlorite solution. The NGM plates (cultured for 2–3 days after seeding) were washed with M9 buffer. All the worms and eggs were collected in a tube, and hypochlorite solution was added to dissolve all worms (except for the eggs) because eggs were protected by the shell. The remaining eggs were washed with M9 buffer. Then, the buffer and eggs were placed in an incubator maintained at 20 °C for 1 day. The L1 stage larvae hatched and then stopped growing in the absence of food. Synchronized L1 worms were collected and poured onto fresh NGM plates coated with *E. coli* OP50. The worms were cultured continuously until they reached target stage. Worms reaching the target stage were collected and washed twice with M9 buffer immediately before the experiments.

### *C. elegans* Loading on Microtopography

Three different strains, wild type (N2), *mec‐4*(*e1339*), and *trp*‐4(*sy695*), were placed on the micropillar arrays submerged in a buffer solution. Two mutant strains with mechanosensory defects were chosen for locomotion comparison with the wild type worms. The washed worms were loaded onto the plasma‐treated platform. Before worm loading, the platform was filled with M9 buffer (10 µL or less). Then, the worms submerged in the tube were collected (≈5 µL or less) and placed on the platform.

### Observations on Guided Locomotion of *C. elegans* and Magnetic Actuation of Micropillars

A video microscope (SV‐55, Sometechvision) was used to observe the locomotion of *C. elegans* in magnetically reconfigurable microarrays. The video was captured at constant time intervals to analyze the changes of head director, body lengths, and locomotion velocity of locomoting *C. elegans*, and magnetically changed angles of micropillars, interpillar spacings, and pillar height. The Voronoi diagram was investigated using ImageJ software.

### Statistical Analysis

Head directors, body lengths, and body widths of *C. elegans* were measured using ImageJ software. This software was also utilized to obtain dimensional parameters of upright and magnetically actuated microbarriers, such as actuation angles, *x*‐ and *y*‐axial interpillar spacings, and pillar height. The values were presented as average ± standard deviation after measuring five different microbarriers. The change of *x*‐ and *y*‐displacements of locomoting *C. elegans* were obtained using Kinovea and Tracker software. Locomotion velocity was calculated by dividing distance equal to two body lengths by time required for locomotion. The velocity values were presented as average ± standard deviation after calculating five cases of locomoting worms. The probability (*p*) for locomotion of *C. elegans* was obtained by counting the number of worms implementing locomotion modes, such as sinusoidal behavior, linear translation, navigating movement, circumventing movement, and climbing. The respective counted value was divided by the sum of the number of worms implementing multi‐modal locomotion modes in a microtopography. After multiplying 100, *p* for respective locomotion mode was denoted by histograms. All these statistical data were indicated in Tables and Figures.

## Conflict of Interest

The authors declare no conflict of interest.

## Supporting information

Supporting Information

Supplemental Video 1

Supplemental Video 2

Supplemental Video 3

Supplemental Video 4

Supplemental Video 5

## Data Availability

The data that support the findings of this study are available from the corresponding author upon reasonable request.
