# Supplementary material for: Multi‐Modal Locomotion of Caenorhabditis elegans by Magnetic Reconfiguration of 3D Microtopography
Source: Adv Sci (Weinh). 2022 Oct 31;9(36):2203396. doi: 10.1002/advs.202203396 (PMC9798981; doi:10.1002/advs.202203396)
Supplement: Supplementary file 1 — Supporting Information [file ADVS-9-2203396-s005.pdf]

ADVANCED SCIENCE
Open Access

Supporting Information

for *Adv. Sci.*, DOI 10.1002/advs.202203396

Multi-Modal Locomotion of *Caenorhabditis elegans* by Magnetic Reconfiguration of 3D Microtopography

Jeong Eun Park, Sunhee Yoon, Jisoo Jeon, Chae Ryean Kim, Saebohm Jhang, Tae-Joon Jeon, Seung Goo Lee, Sun Min Kim* and Jeong Jae Wie**

Supporting Information

Multi-Modal Locomotion of *Caenorhabditis Elegans* by Magnetic reconfiguration of Three-Dimensional Microtopography

Jeong Eun Park,[1,2] *Sunhee Yoon,*[3] *Jisoo Jeon,*[2] *Chae Ryean Kim,*[4] *Saebohm Jhang,*[2] *Tae-Joon Jeon,*[3] *Seung Goo Lee,*[4*] *Sun Min Kim,*[3,5*] *Jeong Jae Wie*[6,7,8,9*]

[1]The Research Institute of Industrial Science, Hanyang University, Seoul 04763, Republic of Korea

[2]Program in Environmental and Polymer Engineering, Inha University, Incheon 22212, Republic of Korea

[3]Department of Biological Sciences and Bioengineering, Inha University, Incheon 22212, Republic of Korea

[4]Department of Chemistry, University of Ulsan, Ulsan 44602, Republic of Korea

[5]Department of Mechanical Engineering, Inha University, Incheon 22212, Republic of Korea

[6]Department of Organic and Nano Engineering, Hanyang University, Seoul 04763, Republic of Korea

[7]Human-Tech Convergence Program, Hanyang University, Seoul 04763, Republic of Korea

[8]Department of Chemical Engineering, Hanyang University, Seoul 04763, Republic of Korea

[9]Institute of Nano Science and Technology, Hanyang University, Seoul 04763, Republic of Korea

* Corresponding author e-mail: lees9@ulsan.ac.kr (S. G. Lee), sunmk@inha.ac.kr (S. M. Kim), jjwie@hanyang.ac.kr (J. J. Wie).

Video S1: Upright microbarrier-guided linear translation of *C. elegans*

Video S2: Reversible magnetic actuations of micropillar Arrays at increasing and decreasing Magnetic field from 0.05 to 0.6 T and vice versa

Video S3: Multi-modal locomotion on twisted/pairwise assembled microtopography at increasing magnetic field from 0.05 to 0.6 T

Video S4: Multi-modal locomotion on twisted/bent/connectively assembled microtopography at increasing magnetic field from 0.05 to 0.6 T

Video S5: Linear translation on twisted/pairwise assembled microtopography with three different *C. elegans* strains

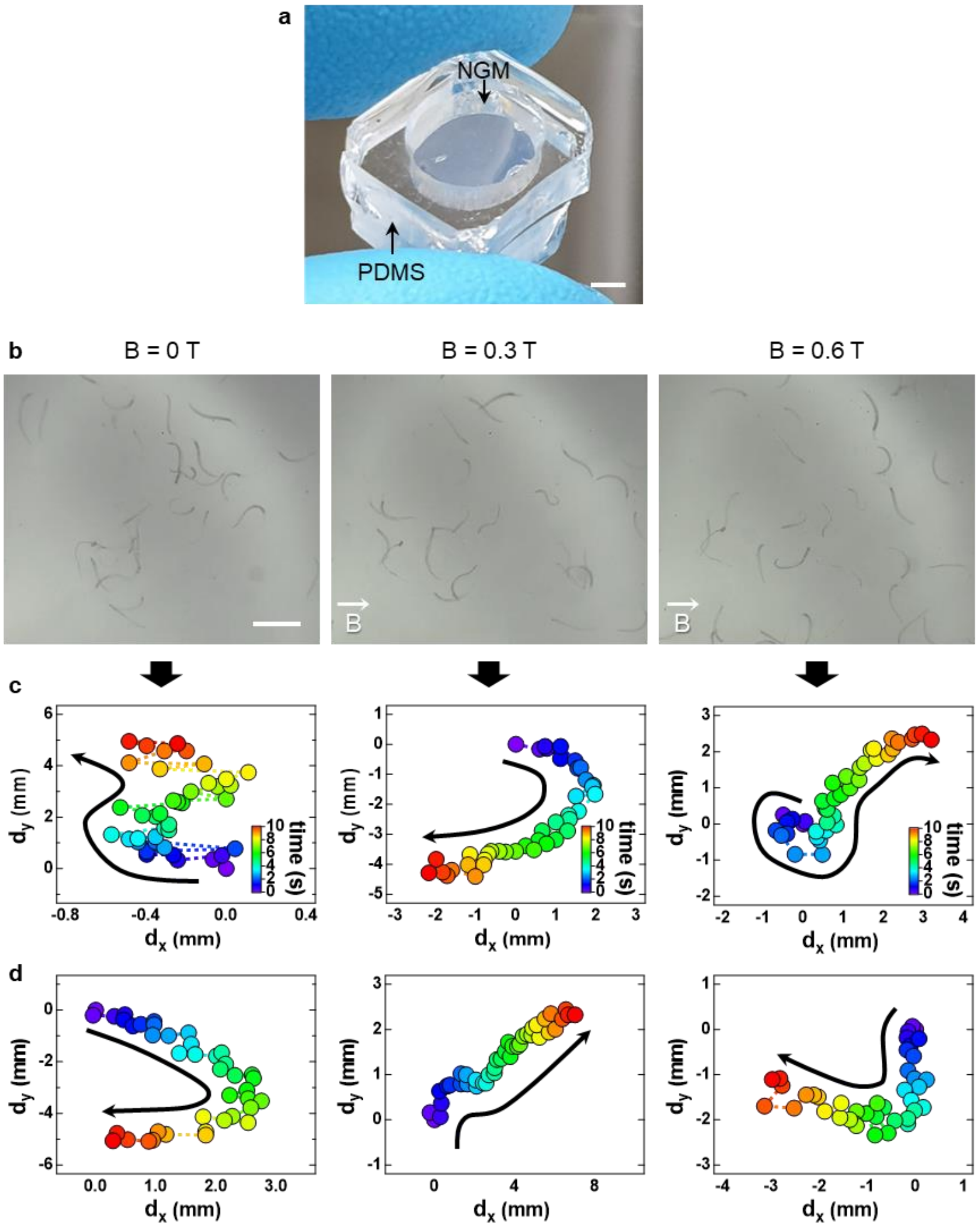

Figure S1. Sinusoidal behavior of *C. elegans* in barrier-free chamber under applying magnetic field. a) NGM plate and PDMS bath are employed as transparent environment to observe movement of *C. elegans* with transparent body. b) There is no preferred direction of progress along the magnetic field axis (scale bars: a) 2, and b) 1 mm). Here, each magnetic field was applied from left to right for 1 min, and then, increase or decrease, respectively. We also observed trajectories of c,d) two randomly selected worm with sinusoidal behavior at each magnetic field.

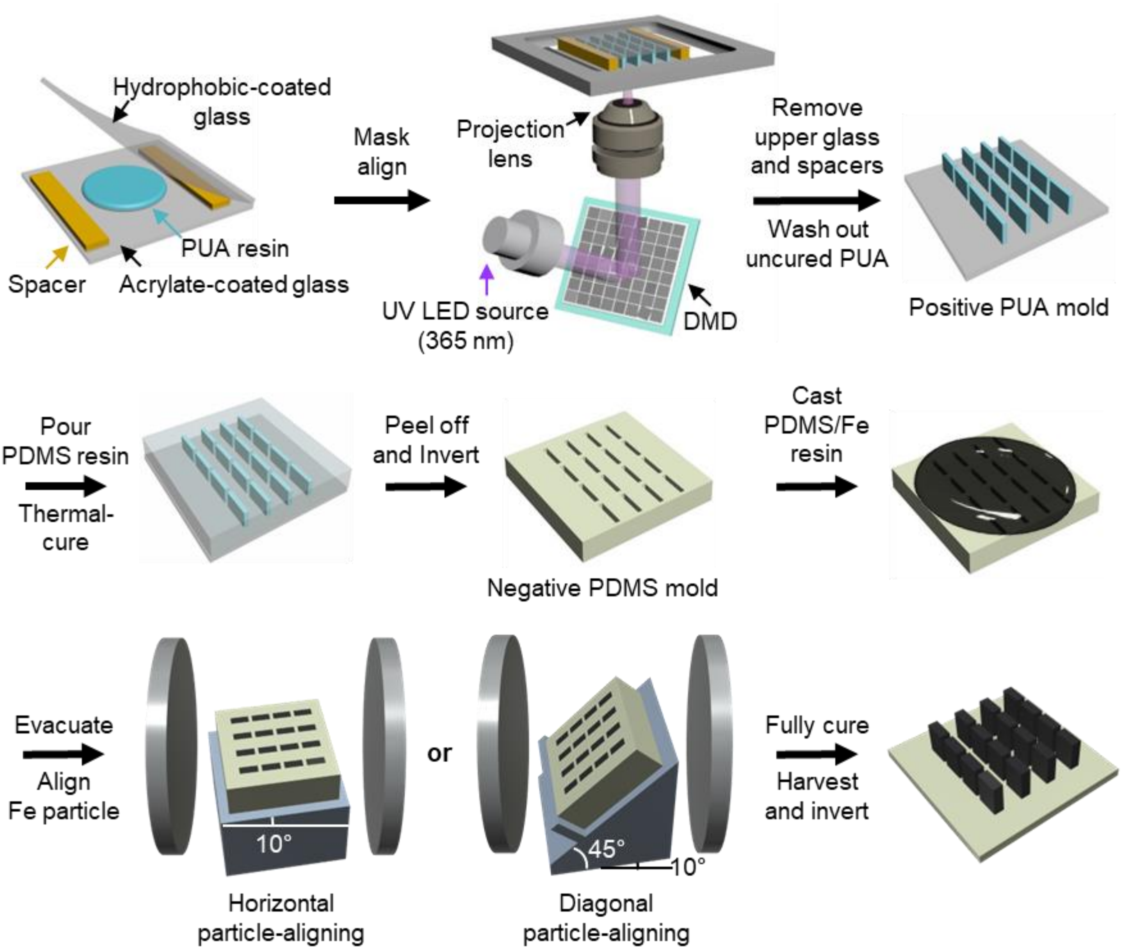

Figure S2. Fabrication schemes of micropillar arrays for three-dimensional magnetic actuation. DMD is employed to prepare the master mold with desired design. Mixture of iron (Fe) particles and prepolymer PDMS resin is poured on negative mold with evacuation, and arrangement of Fe particles is designed along horizontal or diagonal (45° angled) axes by linear magnetic field generated by two permanent magnets. After curing, final product of micropillar arrays is harvested from mold.

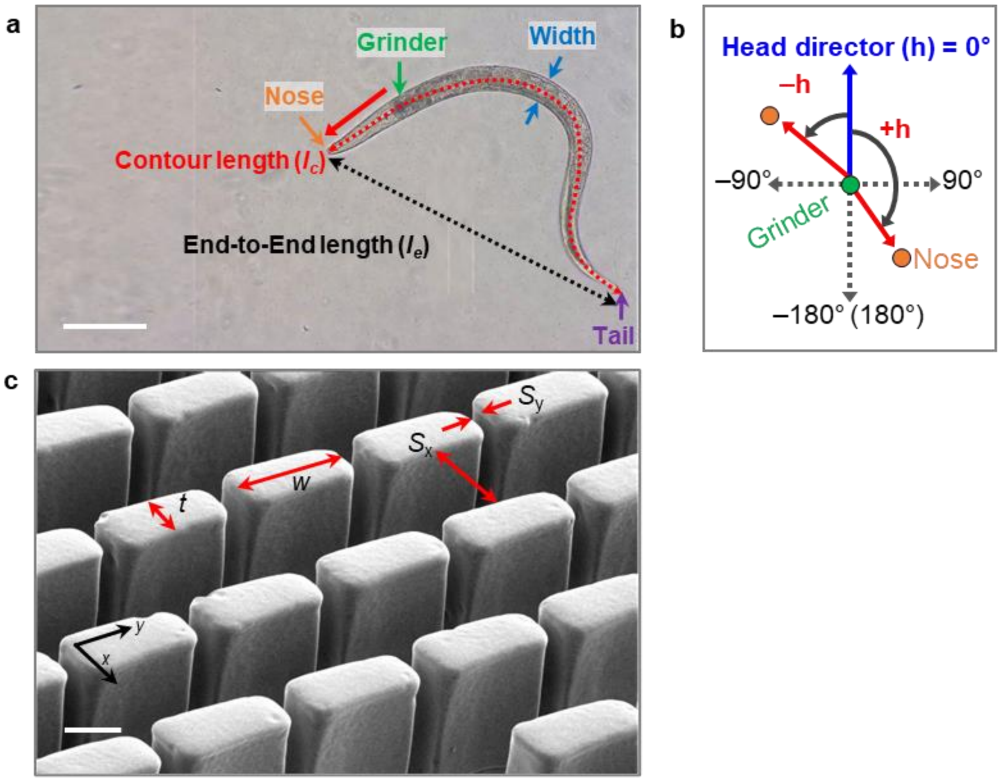

Figure S3. Wild type *C. elegans*. a) Grinder, nose and tail of *C. elegans* are identified in the optical image. Contour length, l_c, and end-to-end length, l_e, are measured based on distance from nose to tail. b) Head director, *h*, of *C. elegans* is defined based on direction from grinder to nose. c) Tilt-view scanning electron microscopy image of periodically arranged upright pillar arrays. Width is $w = 110$ µm, thickness is $t = 250$ µm, spacing along *x*-axis is $S_x = 50$ µm, and spacing along *y*-axis is $S_y = 210$ µm (scale bars: 200 µm).

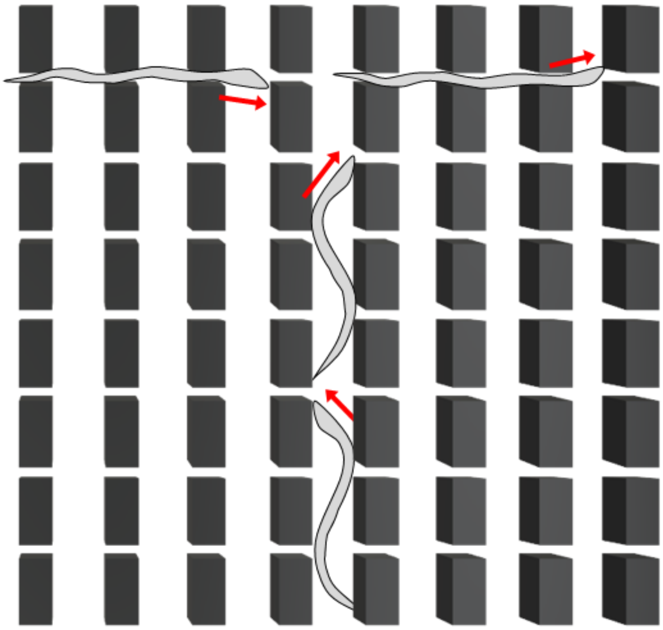

Figure S4. Scheme of biaxial linear translation of *C. elegans* among gaps of upright rectangular micropillar arrays.

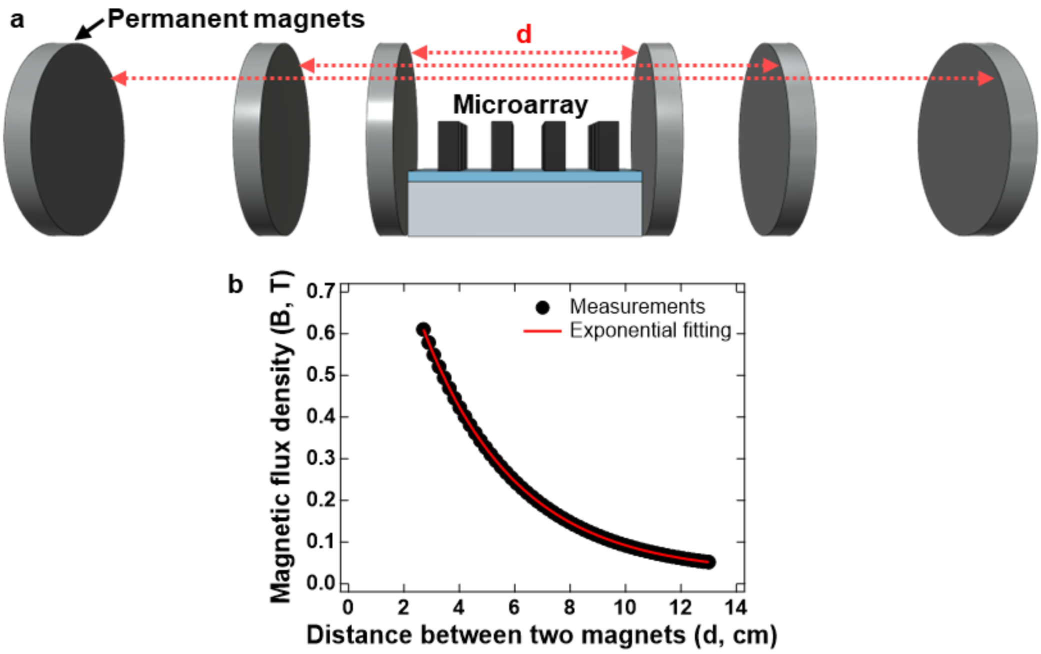

Figure S5. Magnetic field system. a) Microarray sample is placed between two permanent magnets b) where gap distance *d* relates to applied magnetic flux density *B*.

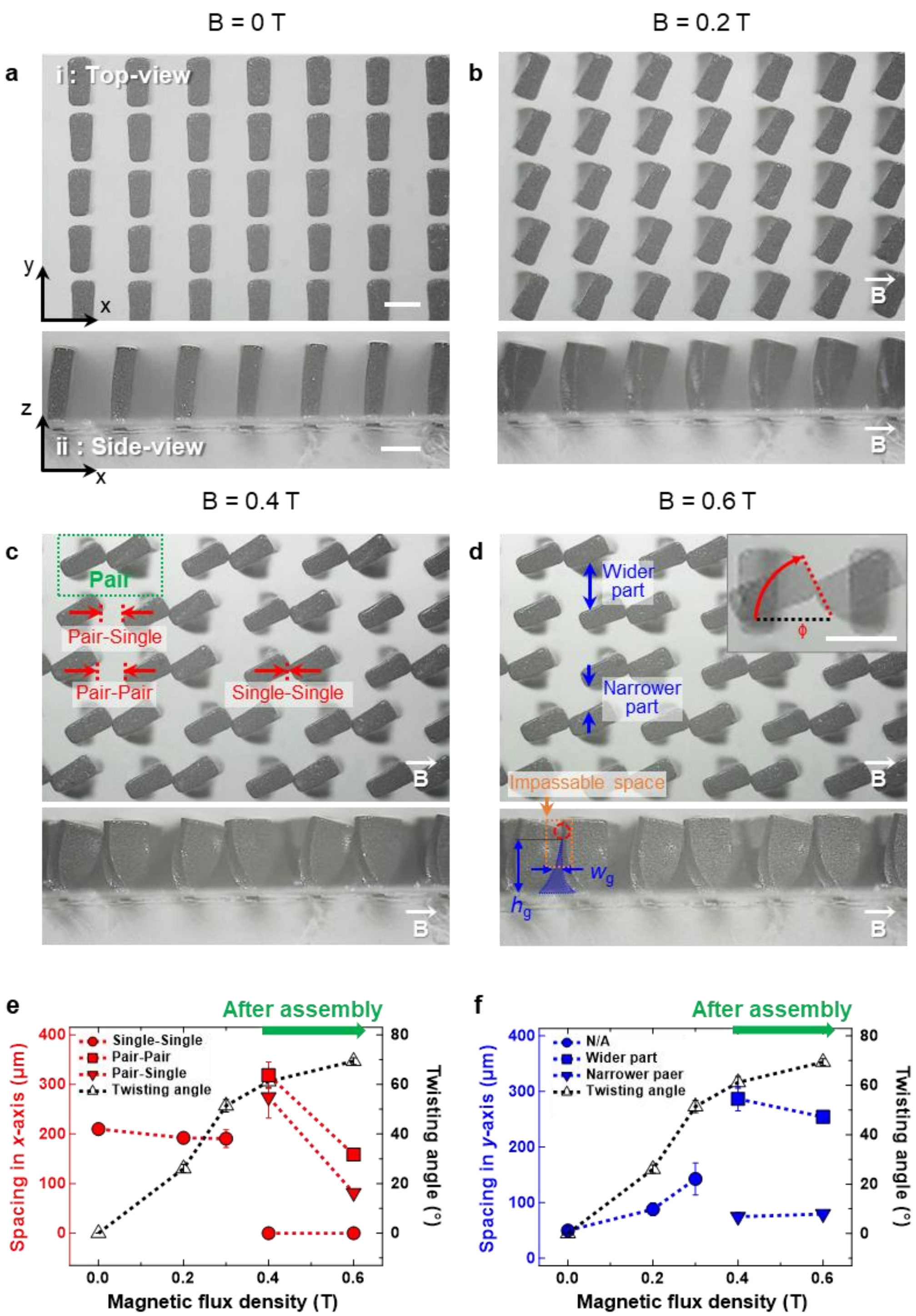

Figure S6. Twisting and pairwise assembly of micropilalr arrays. (i) Top view and (ii) side view images at a) B = 0 T, b) 0.2 T, c) 0.4 T, and d) 0.6 T (scale bars: 200 μm). e) Spacing along x-axis and f) y-axis change according to magnetic actuation states at increasing magnetic flux density. In d-ii), gap between the assembled micropillars is blocked (red mark) and narrowed (blue mark) at the top and base, respectively. Here, height of gap (h_g) and width of gap (w_g) are measured as ~284.2 and ~40.4 μm. Half of gap (organe mark) was considered impassable to *C. elegans*.

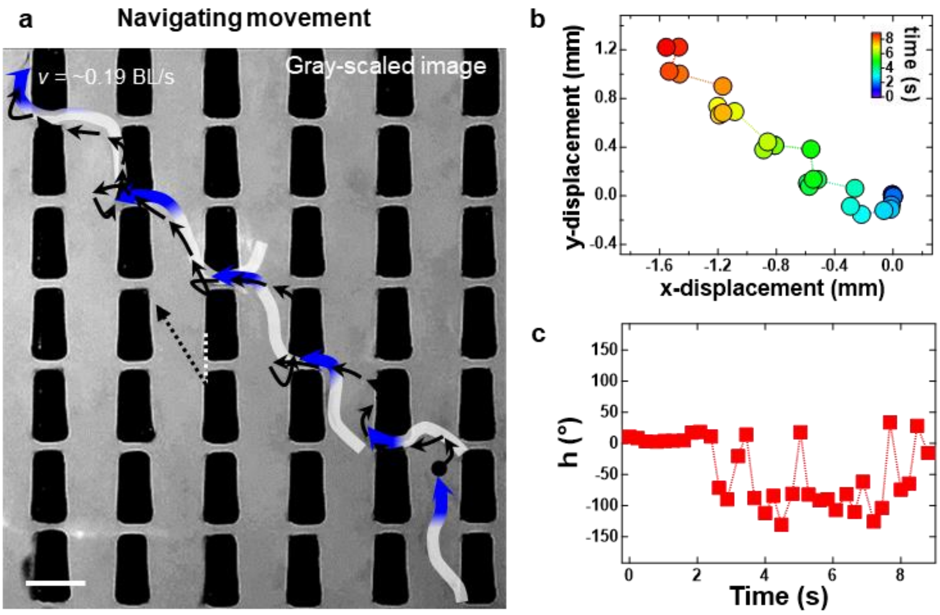

Figure S7. Diagonal navigating movement of *C. elegans*. a) Optical image of snapshots indicates *h* and the body of *C. elegans*. The progress velocity is 0.19 ± 0.05 BL s^{-1}. b) Coordinate graph. c) Oscillation graph of *h*. (scale bar: 200 μm).

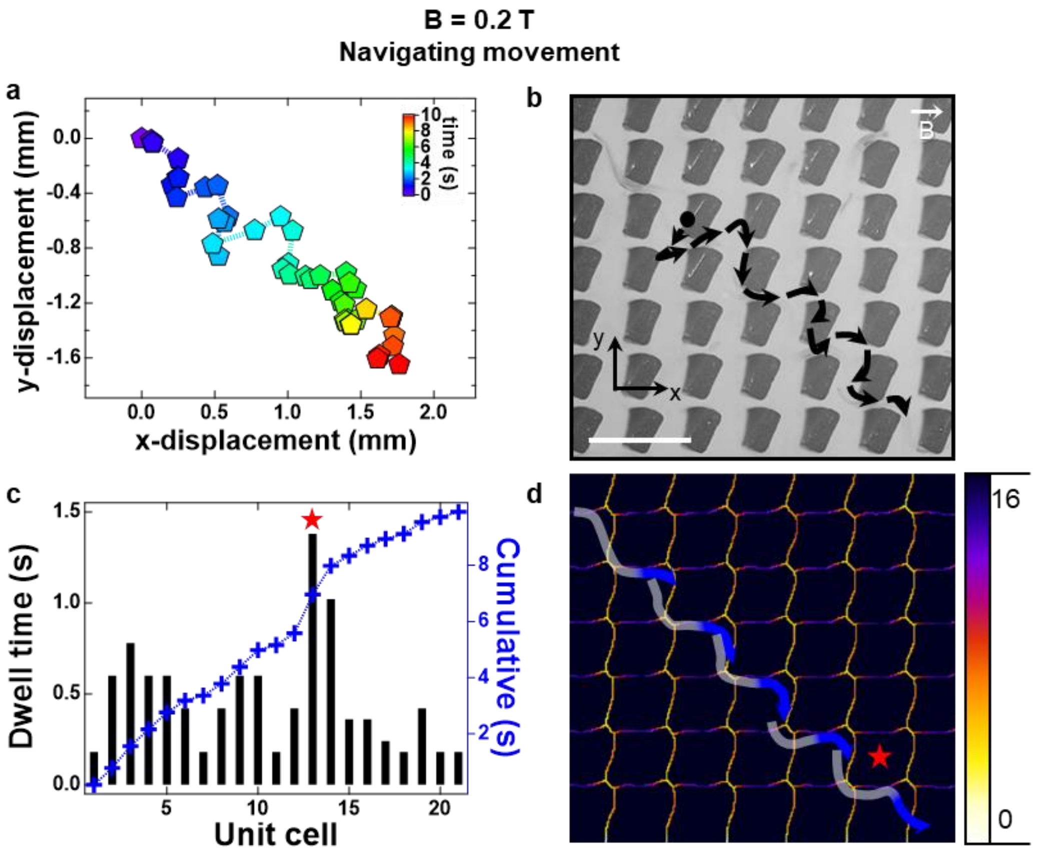

Figure S8. Navigating movement on twisted and pairwise-assembled micropillar arrays. a) Coordinates, b) optical image (scale bar: 500 μm), and c) dwell time analyzed by d) Voronoi image of navigating movement at B = 0.2 T. Average dwell time at single unit cell is 0.5 ± 0.3 s.

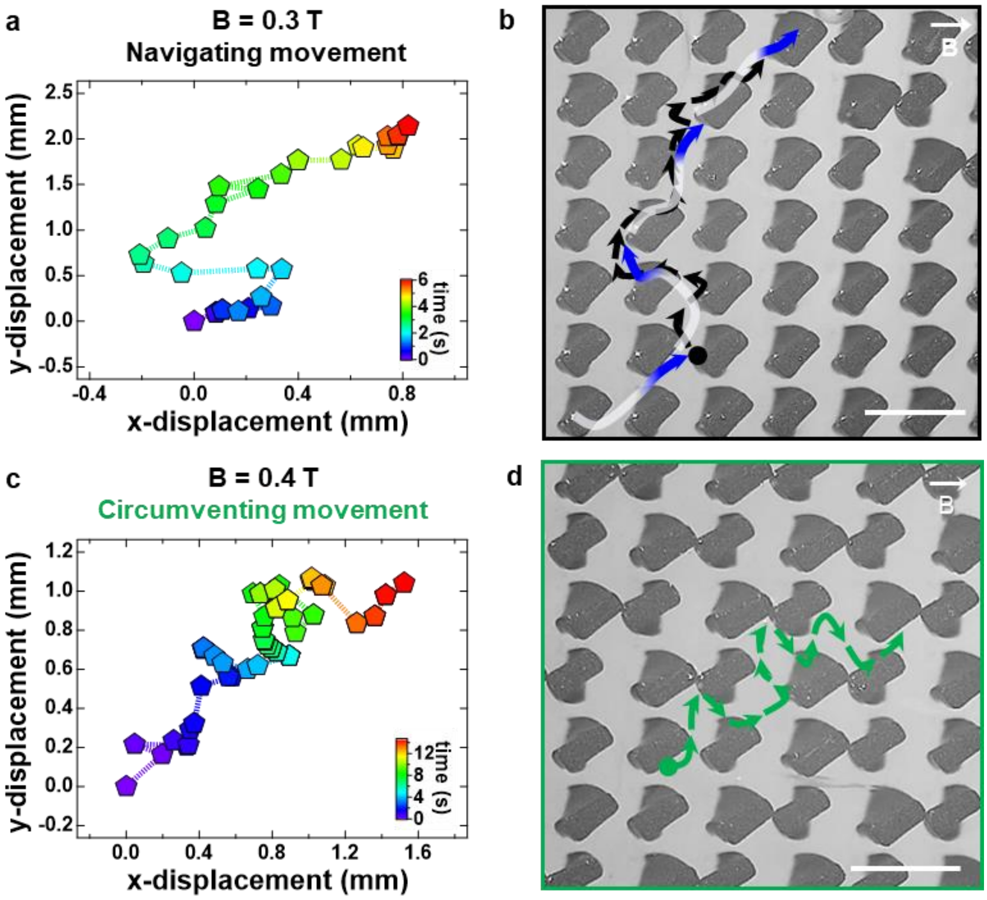

Figure S9. Locomotive modes on twisted and pairwise-assembled micropilalr arrays. a,c) Coordinates and b,d) images to indicate a,b) navigating movement at *B* = 0.3 T and c,d) circumventing movement at *B* = 0.4 T, respectively. (scale bars: 500 μm)

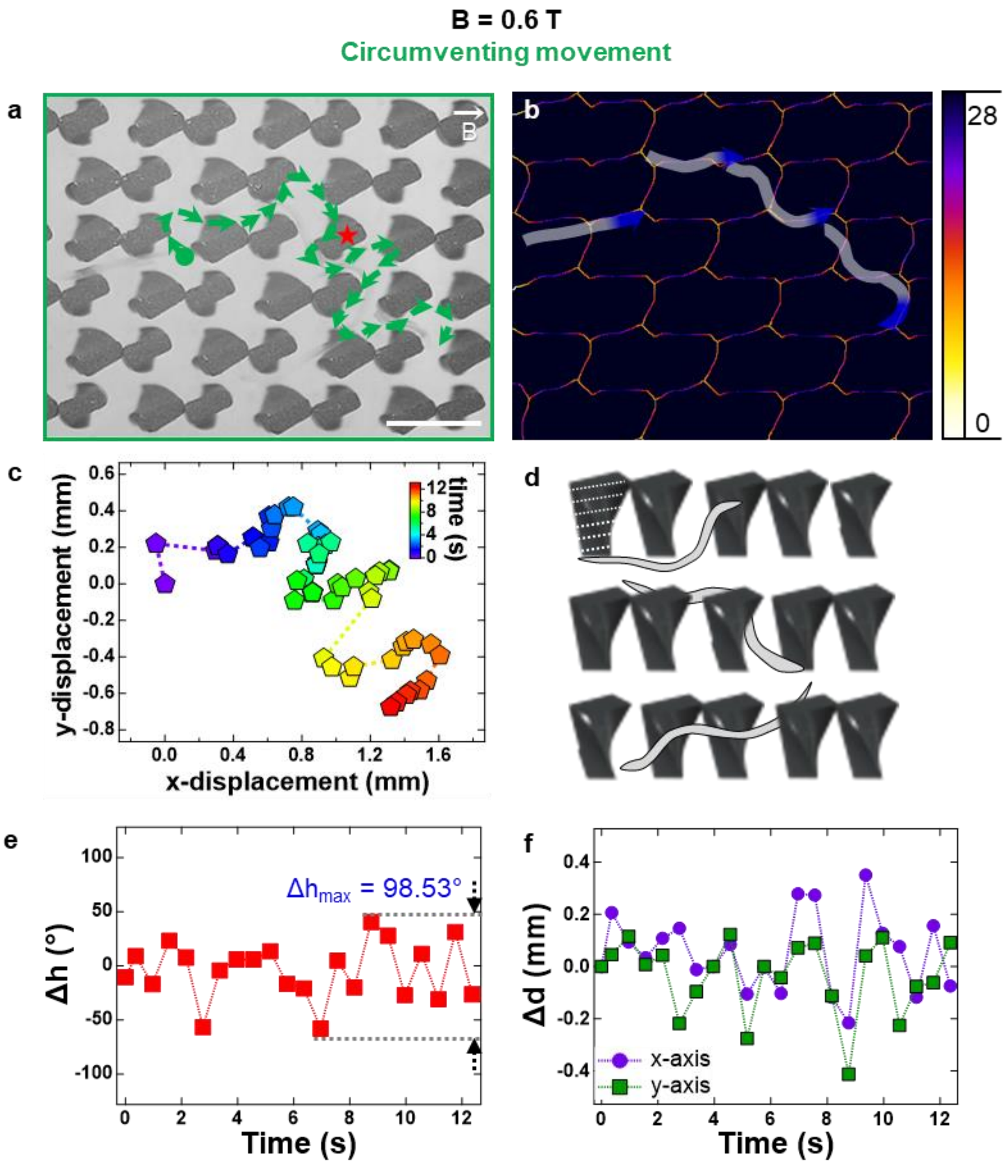

Figure S10. Additional analysis of circumventing movement at B = 0.6 T. a) Optical image (scale bar: 500 μm), b) Voronoi regimes (averaged dwell time at single unit cell = 0.7 ± 0.4 s), c) trajectory, and d) scheme of circumventing movement on twisted and pairwise-assembled micropillars. e) Intrinsic property of sinusoidal heading can be confirmed by head director deviation (Δh) and f) displacement deviation (Δd). Head director deviation, Δh, is defined by subtraction from actuation director (a) and head director (h).

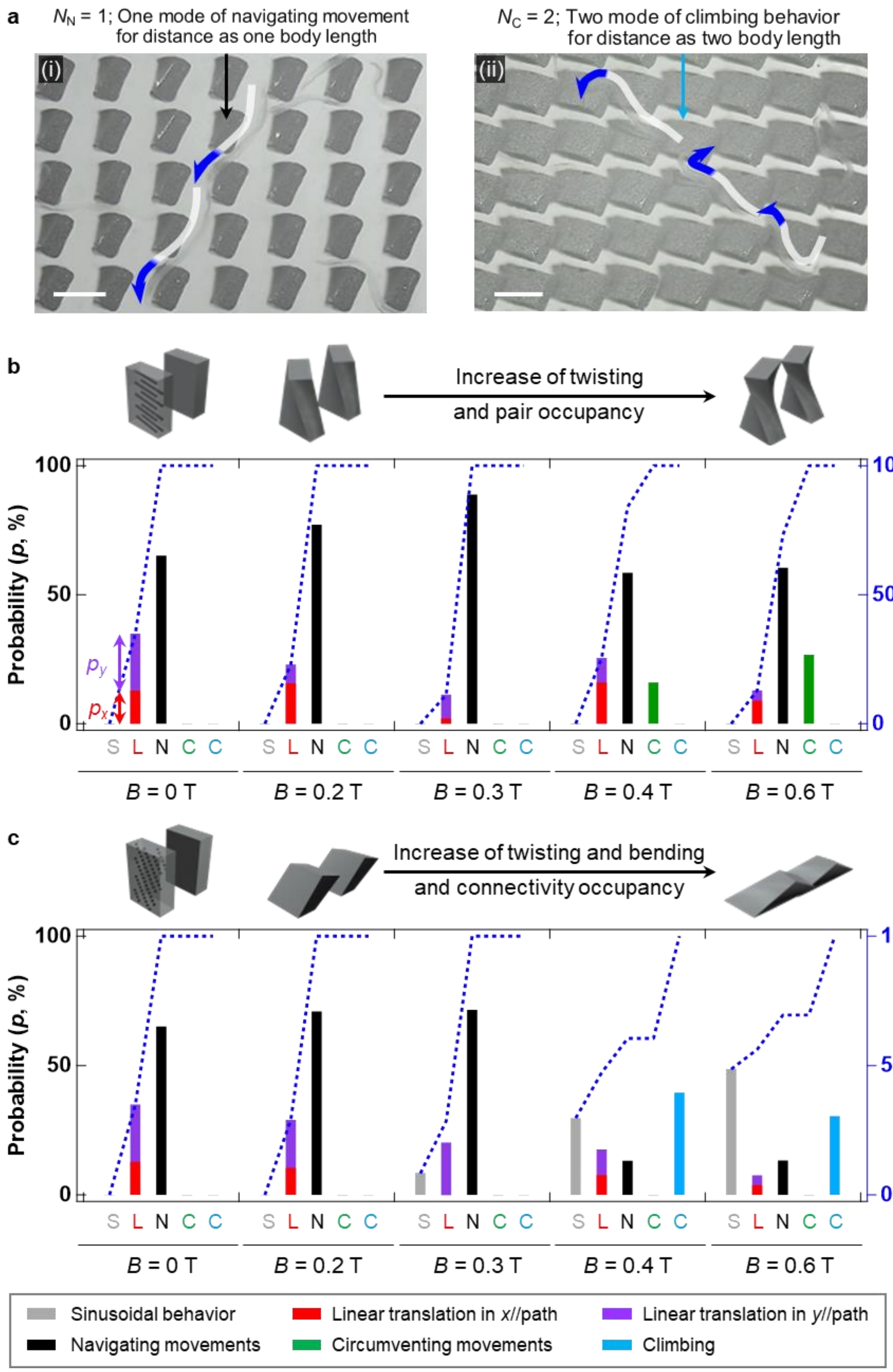

Figure S11. Statistical analysis for physically guided locomotion modes. a) Definition of the number of a mode (N_{mode}) emerged on b) twisted/pairwise assembled and c) twisted/bent/connectively assembled microtopography. Probability p is calculated by dividing N_{mode} by sum of N_{mode} of locomotion modes emerged at each B and multiplying 100. Probability p of sinusoidal behavior, x/y-axial linear translation, navigating movement, circumventing movement, and climbing are denoted by gray, red/purple, black, green, and sky histogram, depending on increase of magnetic flux density, B. (scale bars: 300 μm).

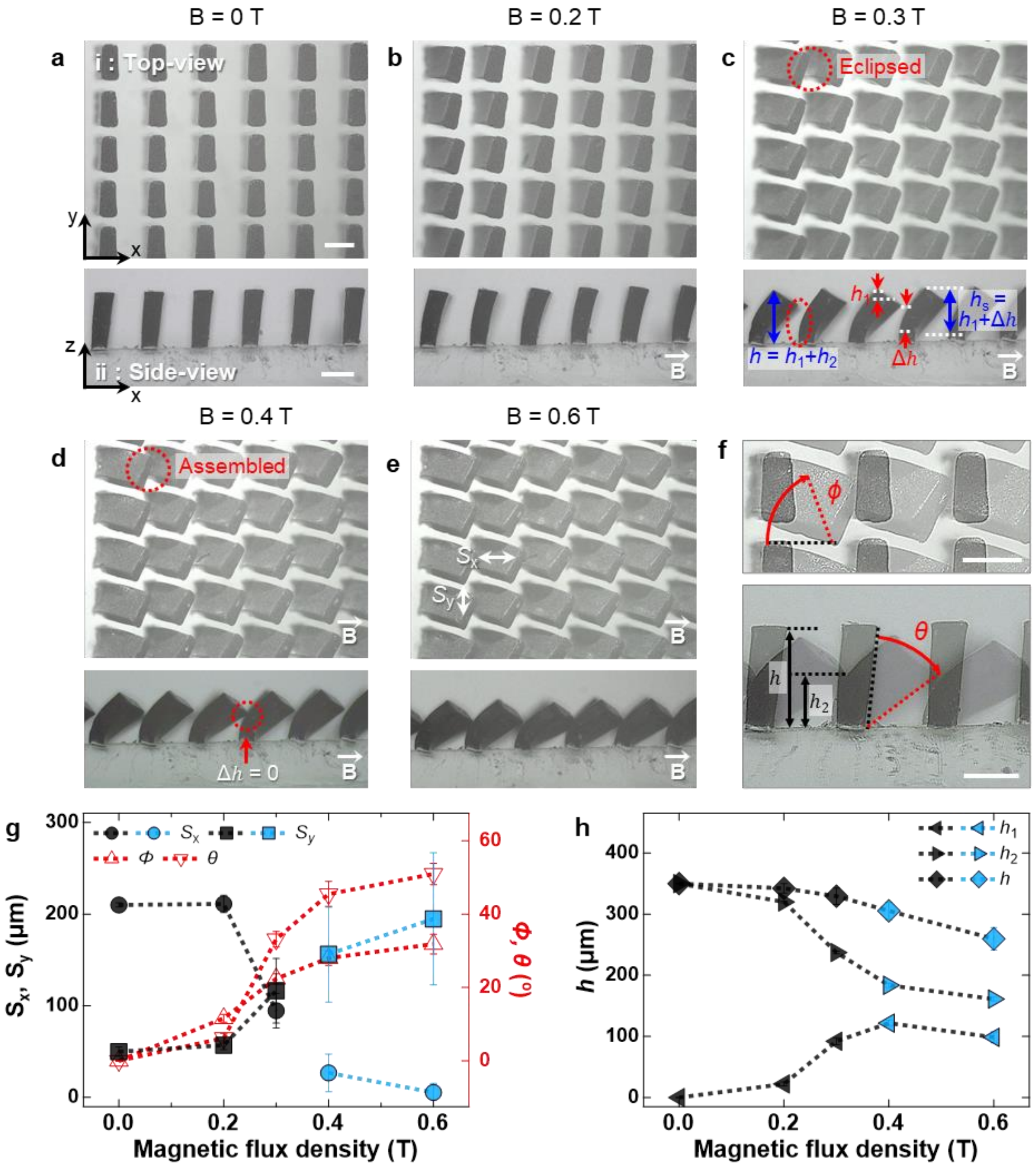

Figure S12. Twisting, bending, and connecitve assembly of micropillar arrays. (i) Top view and (ii) side view images at a) B = 0 T, b) 0.2 T, c) 0.3 T, d) 0.4 T, and e) 0.6 T. c) denotes definition of height of bent pillar (h, h_1), height of step, h_s, and height difference, Δh. f) shows definition of twisting angle (ϕ), bending angle (θ), and h and h_2 using images at 0 T and 0.3 T (scale bars: 200 μm). g), h) descibe S_x, S_y, ϕ, θ, h, h_1, and h_2 at increased magnetic flux density.

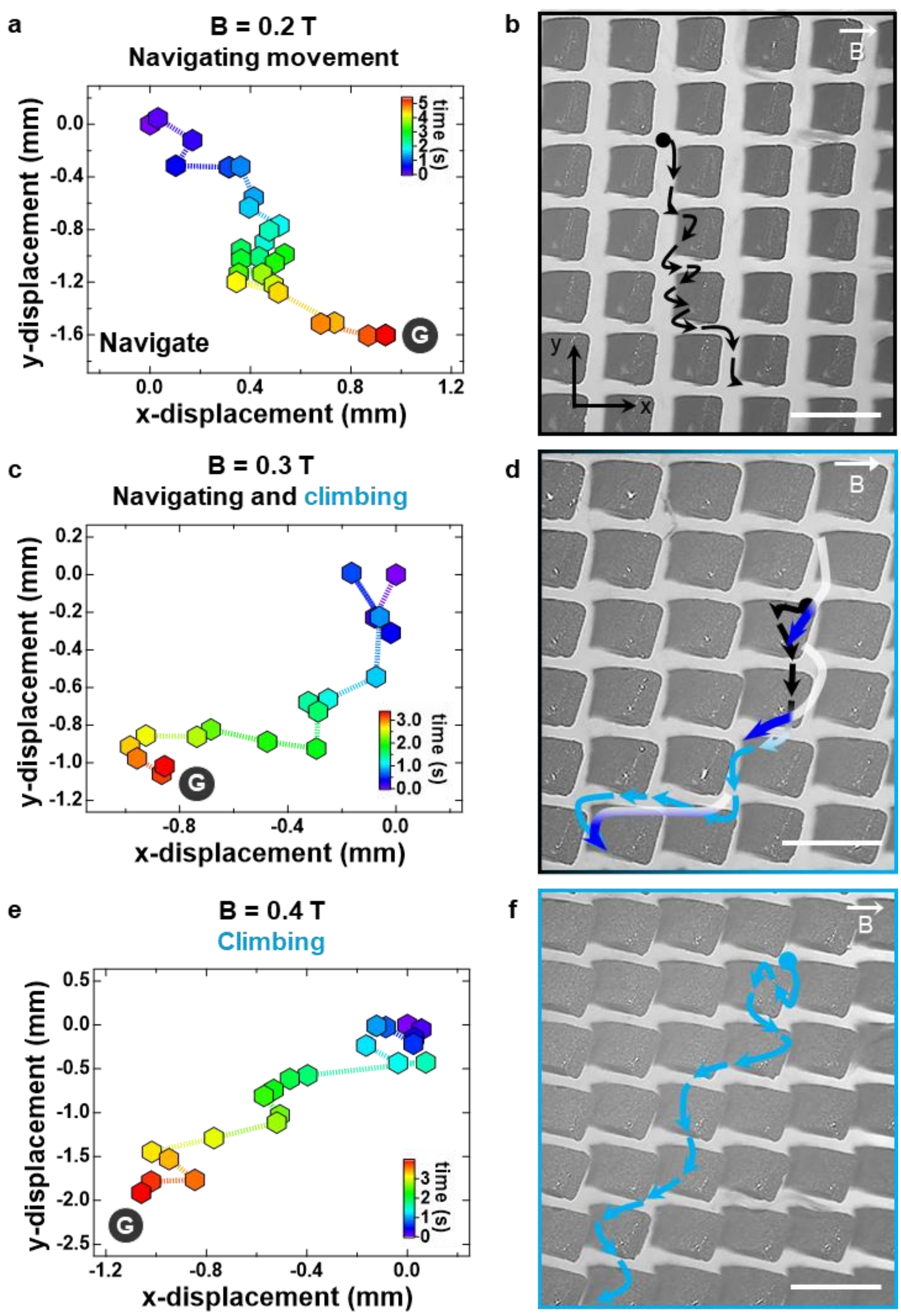

Figure S13. Locomotive modes on twisted and bent micropillar arrays. a,c,e) Coordinates and b,d,f) optical images of a,b) navigating movement at B = 0.2 T, c,d) hybrid of navigating and climbing at B = 0.3 T, and e,f) climbing at B = 0.4 T. (scale bars: 500 μm).

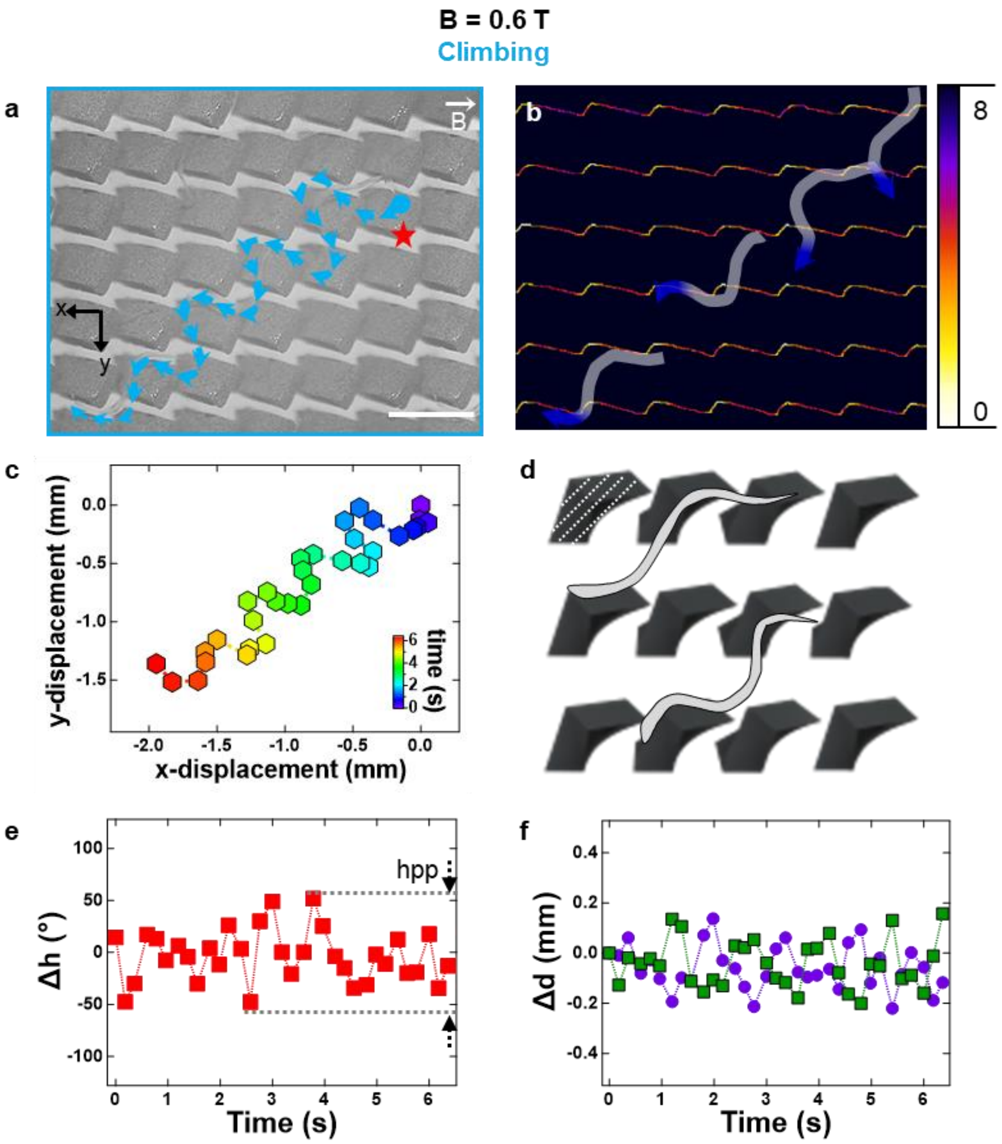

Figure S14. Additional analysis of climbing behavior at B = 0.6 T. a) Optical image (scale bar: 500 μm), b) Voronoi regimes, c) trajectory, and d) scheme of climbing behavior on twisted and bent micropillars. e) Intrinsic property of sinusoidal heading can be confirmed by head director deviation (Δh) and f) displacement deviation (Δd). Head director deviation, Δh, is defined by subtraction from actuation director (a) and head director (h).

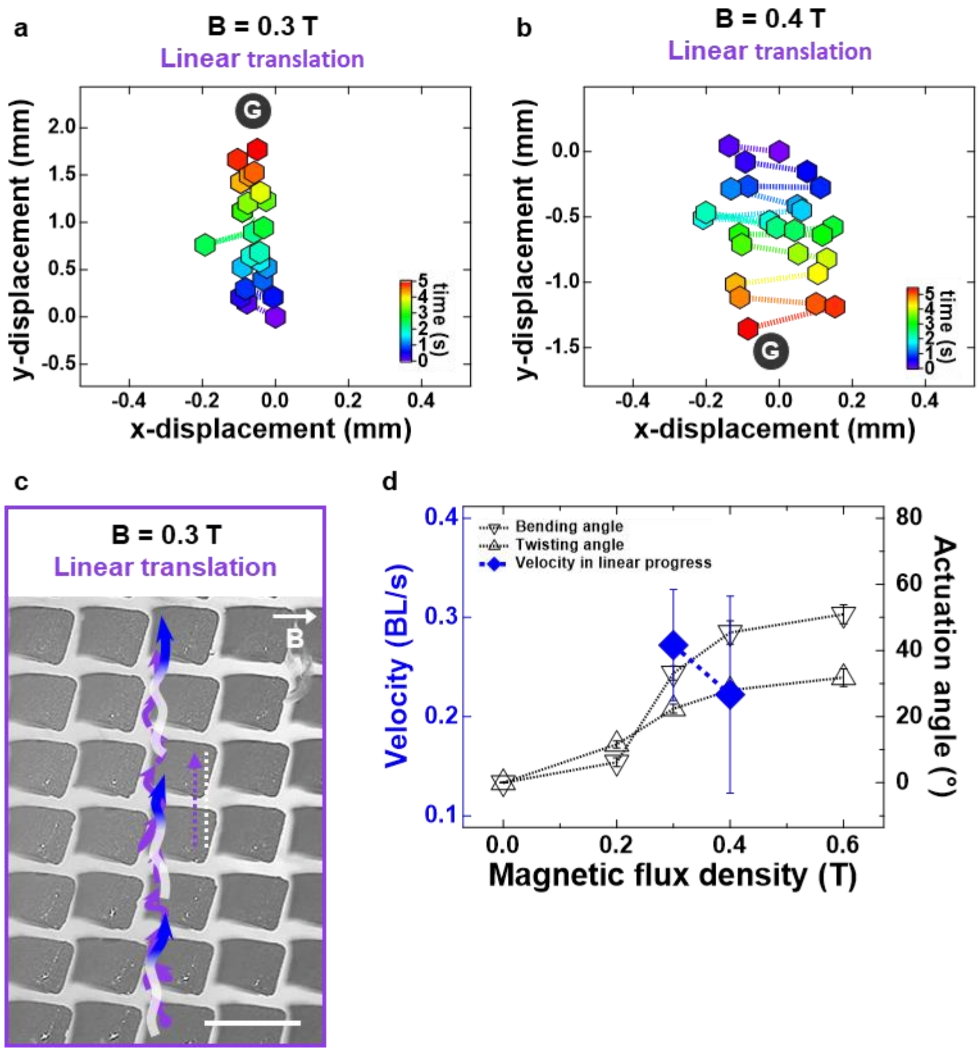

Figure S15. Coordinate graphs of linear translation. Under magnetic field of a) 0.3 T and b) 0.4 T, micropillars are twisted and bent, forming narrow pathways for linear translation of *C. elegans*. c) Optical image denotes linear translation at B = 0.3 T. (scale bar, 500 μm). d) Velocity of *C. elegans* on twisted and bent pillar arrays.

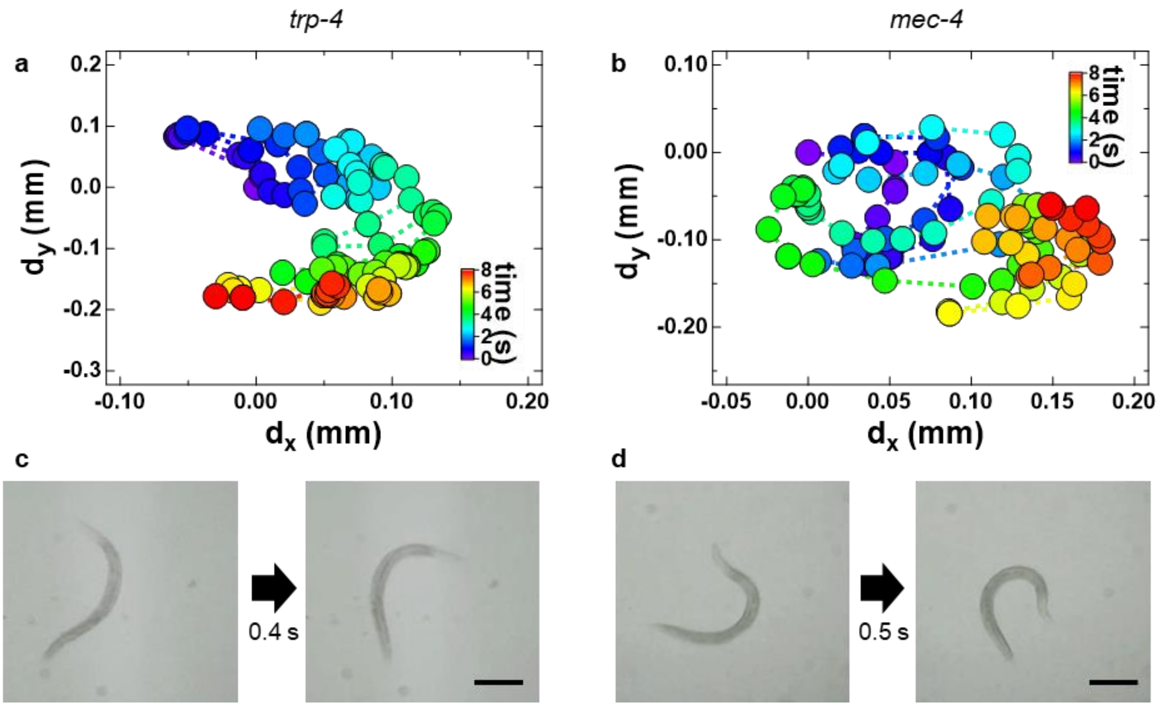

Figure S16. Sinusoidal behavior in bath without microtopography. a) Head/tail mutant (*trp-4(sy695)*) and b) body mutant (*mec-4(e1339)*). c), d) show transition of c-shape body that defines bending velocity, $v_{bending}$ (scale bars: 200 μm).

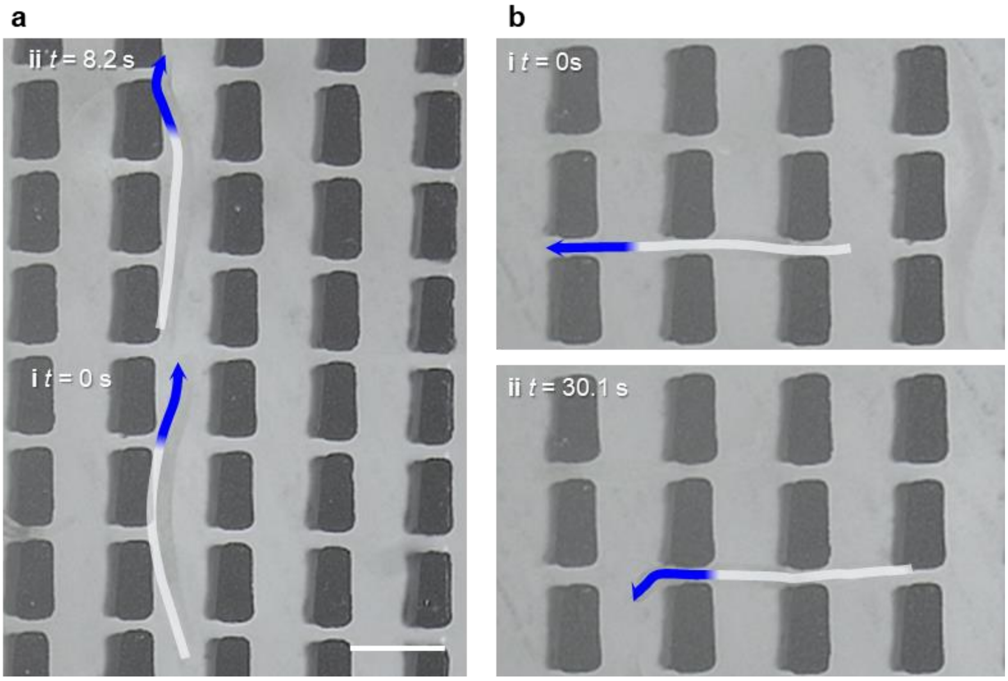

Figure S17. Linear translation of head/tail mutant *trp-4(sy695)*. a) +*y*-direction and b) −*x*-direction of adult *trp-4(sy695)* on microtopography with upright micropillar arrays. Body length and width are 1186.1 and 77.4 µm, respectively. In case of *x*-axis pathways, relatively fat *trp-4(sy695)* is trapped between two upright micropillars with S_y of 50 µm (scale bar: 300 µm).

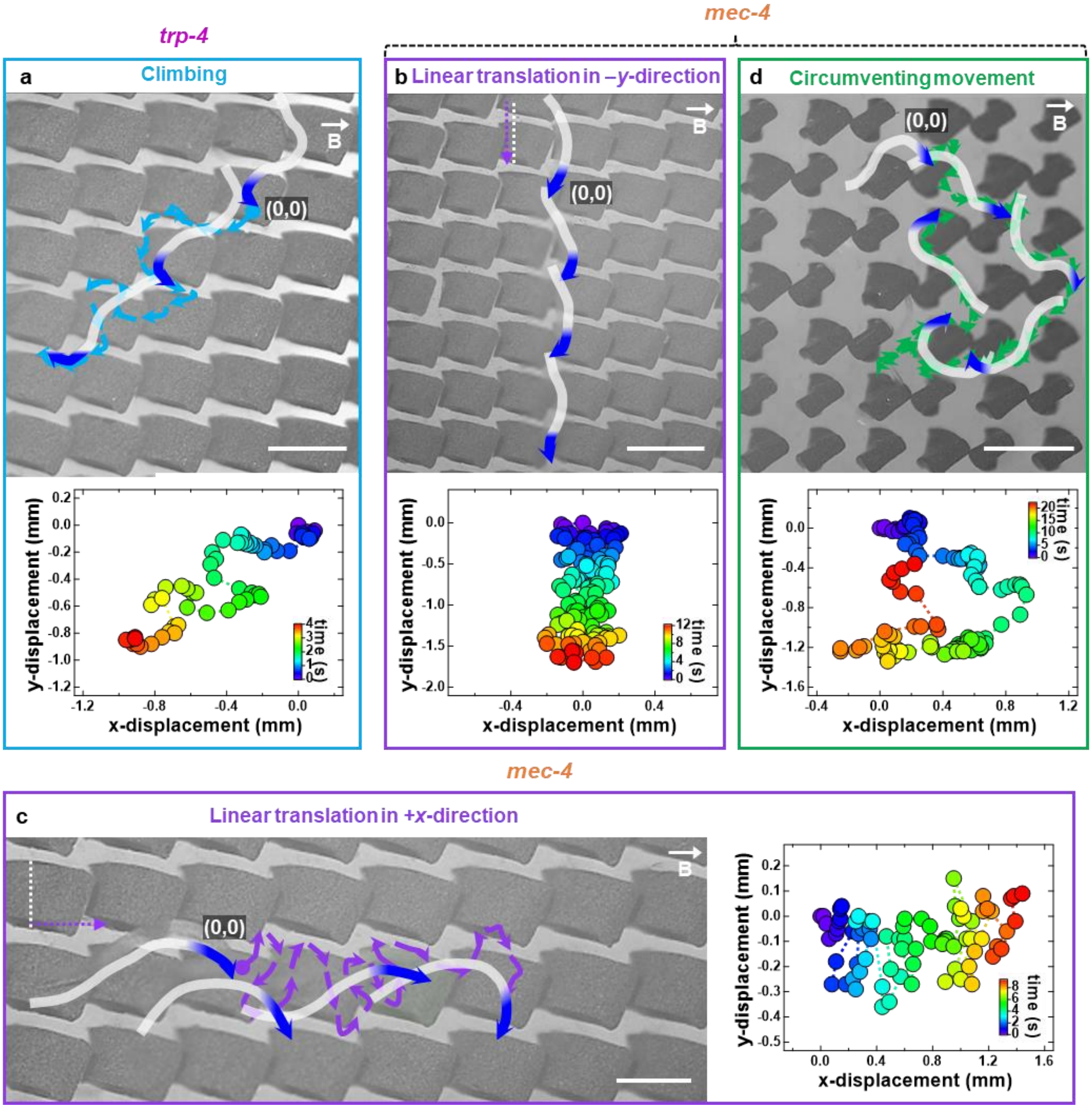

Figure S18. Locomotive modes of *trp-4* and *mec-4*. a) Climbing on twisted and bent micropillars. Linear translation b) in −*y* direction and c) +*x* direction on twisted and bent micorpillars. d) Circumventing movement on twisted and pairwise-assembled micropillars. Scale bars are in a), b), and d) and c) are 500 and 300 μm, respectively.

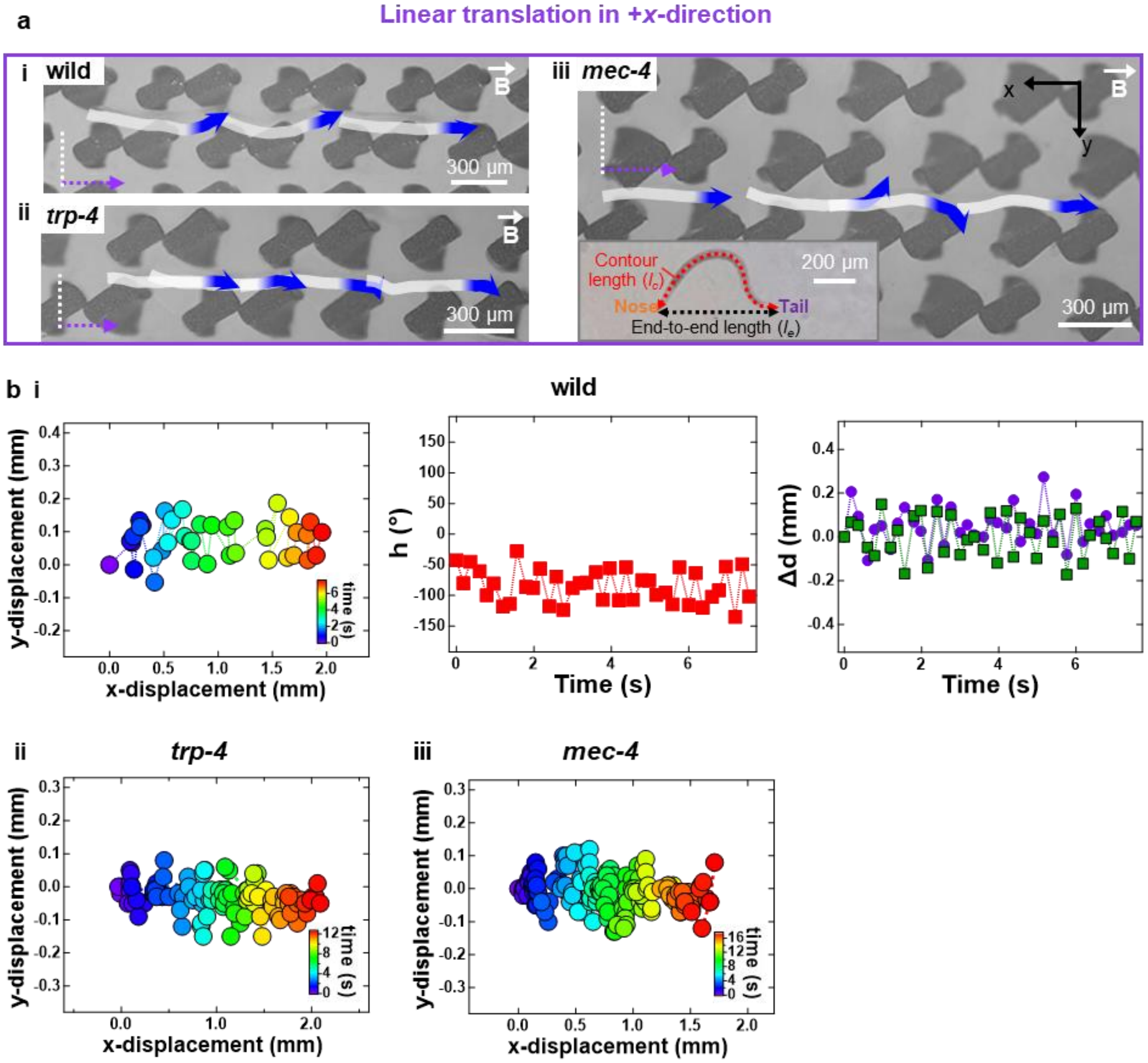

Figure S19. Linear translation in +*x*-direction of *C. elegans* on twisted and pairwise-assembled micropillars. a) Optical images and b) analysis for behaviors of (i) wild, (ii) *trp-4* mutant, and (iii) *mec-4* mutant.

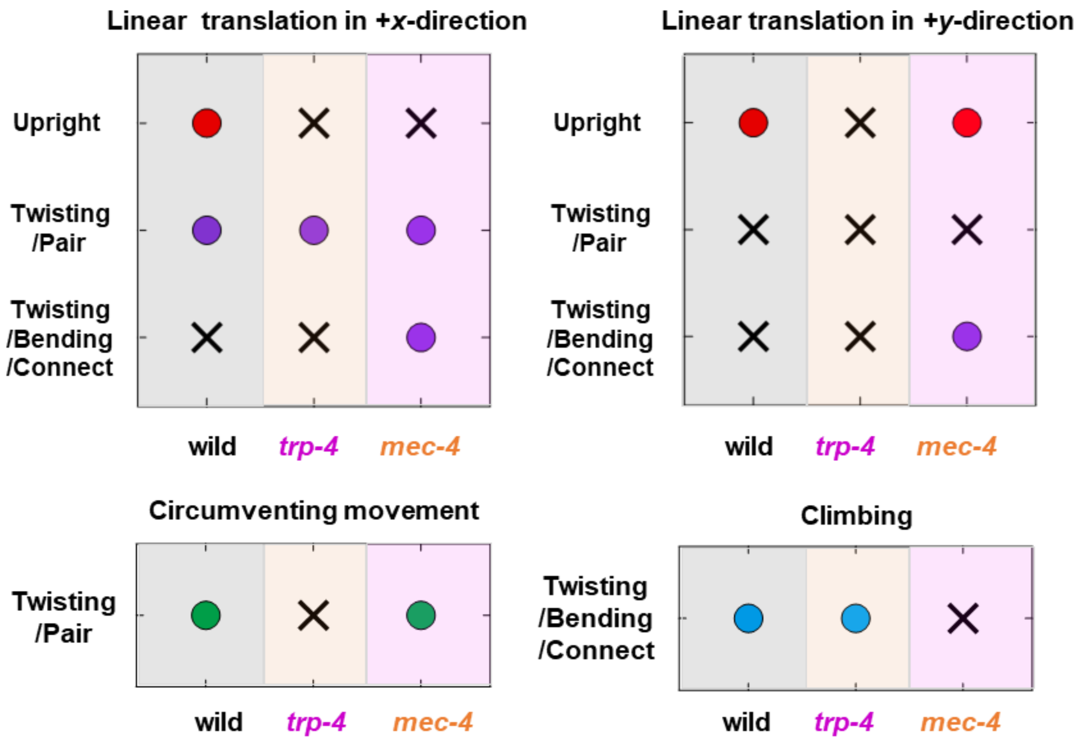

Figure S20. Phase diagram of locomotions programed by microtopography. Wild type, *trp-4* mutant, and *mec-4* mutant are compared. The average contour lengths of each strain are 749, 770, and 694 µm, respectively.

Table S1. Maximum difference between head director ($\Delta \boldsymbol{h}_{max}$) of *C. elegans* on 3D microtopography

**Behaviors**	**Linear translation**			**Circumventing movement**	**Climbing**
**Microtopography**	Upright pillars		Twisting/ Pairwise assembly	Twisting/ Pairwise assembly	Twisting/ Bending actuation
	Narrow path (//long axis)	Wide Path (//short axis)			
**$\Delta \boldsymbol{h}_{max}$ (°)**	87.88	36.30	107.26	106.15	182.29

Table S2. Magnetic actuation degree of micropillars according to increased magnetic flux density

	**Twisting/Pairwise assembly**	**Twisting/Bending actuation/Connective assembly**	
Magnetic flux density (T)	Twisting angle (°)	Twisting angle (°)	Bending angle (°)
0.2	26.1 ± 1.7	11.6 ± 1.1	6.2 ± 1.3
0.3	51.4 ± 2.5	22.4 ± 1.4	33.2 ± 2.1
0.4	61.1 ± 2.4	28 ± 2.0	45.4 ± 3.5
0.6	69.4 ± 1.4	31.8 ± 2.7	51.0 ± 2.9

Table S3. Variation of pillar spacing along *x*-axis (S_x) and *y*-axis (S_y) caused by twisting/pairwise assembly and twisting/bending actuation/connective assembly at increased magnetic flux density, *B*

	**Upright state**	**Twisting actuation**		**Twisting/Pairwise assembly**		
	B = 0 T	B = 0.2 T	B = 0.3 T		B = 0.4 T	B = 0.6 T
**S_x (µm)**	210 ± 2.2	192.3 ± 5.5	190.7 ± 18.0	Single–Single	0	
				Pair–Pair	318.4 ± 26.4	158.6 ± 8.2
				Pair–Single	274.1 ± 42.0	81.5 ± 0.1
**S_y (µm)**	50 ± 5.2	88.0 ± 11.4	142.6 ± 28.8	Wide part	286.6 ± 21.7	254.2 ± 6.0
				Narrow part	74.7 ± 7.6	79.6 ± 5.5
	**Upright state**	**Twisting/Bending actuation**		**Twisting/Bending actuation/Connective assembly**		
	B = 0 T	B = 0.2 T	B = 0.3 T	B = 0.4 T		B = 0.6 T
**S_x (µm)**	210 ± 2.2	211.1 ± 9.4	94.6 ± 18.7	26.8 ± 20.5		5.4 ± 9.8
**S_y (µm)**	50 ± 5.2	56.9 ± 4.7	116.3 ± 35.2	156.3 ± 52.5		194.9 ± 72.1

Table S4. Length (l) of micropillar, height of bent pillar (h, h_1, and h_2), height difference (Δh), and height of step (h_s) caused by twisting/pairwise assembly and twisting/bending actuation/connective assembly at increased magnetic flux density, B

	**Upright state**	**Twisting actuation**		**Twisting/Pairwise assembly**	
	B = 0 T	B = 0.2 T	B = 0.3 T	B = 0.4 T	B = 0.6 T
**l (μm)**	350				
	**Upright state**	**Twisting/Bending actuation**		**Twisting/Bending actuation/Connective assembly**	
	B = 0 T	B = 0.2 T	B = 0.3 T	B = 0.4 T	B = 0.6 T
**h_1 (μm)**	0	22.2 ± 3.3	92.1 ± 5.6	121.7 ± 4.8	98.6 ± 8.8
**h_2 (μm)**	350.3 ± 2.1	320 ± 4.7	237.3 ± 7.9	183.6 ± 9.5	161.1 ± 9.8
**h (h_1+h_2, μm)**		342.2 ± 8.0	329.4 ± 13.5	305.3 ± 14.3	259.7 ± 18.6
**Δh (μm)**	NA		168.7 ± 42.8	37.8 ± 33.3	11.4 ± 17.6
**h_s (h_1+Δh, μm)**			260.8 ± 48.4	159.6 ± 38.1	101.0 ± 26.4

Table S5. Velocity (BL s^{-1}) of *C. elegans* in linear translations, navigating movement, circumventing movement, and climbing behavior on 3D microtopography

<table>
<tr><th rowspan="2"></th><th rowspan="2" colspan="2"></th><th colspan="5">Magnetic flux density (T)</th></tr>
<tr><th>0</th><th>0.2</th><th>0.3</th><th>0.4</th><th>0.6</th></tr>
<tr><td rowspan="3">Upright state</td><td rowspan="2">Linear translation</td><td>Narrow //x-axis</td><td>0.14 ± 0.09</td><td rowspan="3" colspan="4">NA</td></tr>
<tr><td>Wide //y-axis</td><td>0.26 ± 0.04</td></tr>
<tr><td colspan="2">Navigating movement</td><td>0.19 ± 0.05</td></tr>
<tr><td rowspan="3">Twisting/ Pairwise assembly</td><td colspan="2">Linear translation Narrow //x-axis</td><td rowspan="8">NA</td><td>0.17 ± 0.04</td><td>NA</td><td>0.25 ± 0.06</td><td>0.20 ± 0.02</td></tr>
<tr><td colspan="2">Navigating movement</td><td>0.21 ± 0.03</td><td>0.36 ± 0.06</td><td>0.27 ± 0.11</td><td>0.28 ± 0.05</td></tr>
<tr><td colspan="2">Circumventing movement</td><td colspan="2">NA</td><td>0.11 ± 0.04</td><td>0.09 ± 0.03</td></tr>
<tr><td rowspan="5">Twisting/ Bending actuation/ Connective assembly</td><td rowspan="3">Linear translation</td><td>Narrow //x-axis</td><td>0.22 ± 0.09</td><td rowspan="2" colspan="3">NA</td></tr>
<tr><td>Wide //y-axis</td><td>0.24 ± 0.04</td></tr>
<tr><td>Narrow //y-axis</td><td>NA</td><td>0.27 ± 0.06</td><td>0.22 ± 0.10</td><td>NA</td></tr>
<tr><td colspan="2">Navigating movement</td><td>0.24 ± 0.07</td><td>0.32 ± 0.08</td><td colspan="2">NA</td></tr>
<tr><td colspan="2">Climbing</td><td colspan="2">NA</td><td>0.46 ± 0.10</td><td>0.30 ± 0.13</td></tr>
</table>

Table S6. Length ratio (end-to-end length/contour length, l_e/l_c), bending velocity ($v_{bending}$, BL s^{-1}), and locomotion velocity (v, BL s^{-1}) of three different strains on 3D microtopography

			**wild**	***trp-4***	***mec-4***
		l_c (μm)	1082.4	1112.7	1002.9
**Sinusoidal behavior**		l_e/l_c	0.48	0.56	0.54
		$v_{bending}$	0.82 ± 0.23	0.93 ± 0.06	0.65 ± 0.13
**Linear translation**	**Upright arrays at 0 T**	l_e/l_c	0.95	NA	NA
		v	0.14 ± 0.09		
	**Twisting/Pairwise assembly at 0.6 T**	l_e/l_c	0.95	0.94	0.96
		v	0.20 ± 0.02	0.14 ± 0.01	0.11 ± 0.02
**Circumventing movement at 0.6 T**		l_e/l_c	0.77	NA	0.76
		v	0.09 ± 0.03		0.07 ± 0.04
**Climbing at 0.6 T**		l_e/l_c	0.69	0.73	NA
		v	0.30 ± 0.06	0.31 ± 0.04	
